# A Unique *cis*-Encoded Small Noncoding RNA Is Regulating *Legionella pneumophila* Hfq Expression in a Life Cycle-Dependent Manner

**DOI:** 10.1128/mBio.02182-16

**Published:** 2017-01-10

**Authors:** Giulia Oliva, Tobias Sahr, Monica Rolando, Maike Knoth, Carmen Buchrieser

**Affiliations:** Institut Pasteur, Biologie des Bactéries Intracellulaires, CNRS UMR 3525, Paris, France; Princeton University

## Abstract

*Legionella pneumophila* is an environmental bacterium that parasitizes protozoa, but it may also infect humans, thereby causing a severe pneumonia called Legionnaires’ disease. To cycle between the environment and a eukaryotic host, *L. pneumophila* is regulating the expression of virulence factors in a life cycle-dependent manner: replicating bacteria do not express virulence factors, whereas transmissive bacteria are highly motile and infective. Here we show that Hfq is an important regulator in this network. Hfq is highly expressed in transmissive bacteria but is expressed at very low levels in replicating bacteria. A *L. pneumophila hfq* deletion mutant exhibits reduced abilities to infect and multiply in *Acanthamoeba castellanii* at environmental temperatures. The life cycle-dependent regulation of Hfq expression depends on a unique *cis*-encoded small RNA named Anti-hfq that is transcribed antisense of the *hfq* transcript and overlaps its 5′ untranslated region. The Anti-hfq sRNA is highly expressed only in replicating *L. pneumophila* where it regulates *hfq* expression through binding to the complementary regions of the *hfq* transcripts. This results in reduced Hfq protein levels in exponentially growing cells. Both the small noncoding RNA (sRNA) and *hfq* mRNA are bound and stabilized by the Hfq protein, likely leading to the cleavage of the RNA duplex by the endoribonuclease RNase III. In contrast, after the switch to transmissive bacteria, the sRNA is not expressed, allowing now an efficient expression of the *hfq* gene and consequently Hfq. Our results place Hfq and its newly identified sRNA anti-*hfq* in the center of the regulatory network governing *L. pneumophila* differentiation from nonvirulent to virulent bacteria.

## IMPORTANCE

The abilities of *L. pneumophila* to replicate intracellularly and to cause disease depend on its capacity to adapt to different extra- and intracellular environmental conditions. Therefore, a timely and fine-tuned expression of virulence factors and adaptation traits is crucial. Yet, the regulatory circuits governing the life cycle of *L. pneumophila* from replicating to virulent bacteria are only partly uncovered. Here we show that the life cycle-dependent regulation of the RNA chaperone Hfq relies on a small regulatory RNA encoded antisense to the *hfq*-encoding gene through a base pairing mechanism. Furthermore, Hfq regulates its own expression in an autoregulatory loop. The discovery of this RNA regulatory mechanism in *L. pneumophila* is an important step forward in the understanding of how the switch from inoffensive, replicating to highly virulent, transmissive *L. pneumophila* is regulated.

## INTRODUCTION

In recent years, the discovery of a class of regulatory elements, called small noncoding RNAs (sRNAs) revealed a high complexity of posttranscriptional gene regulation in prokaryotes and eukaryotes (1). sRNAs were reported to exert a wide range of cellular functions in bacterial physiology, in which rapid and fine-tuned adaptations in response to environmental changes are required (2, 3). sRNAs are classified as *cis*- or *trans*-encoded sRNAs that modulate gene expression through complementarity to their adjacent or distant mRNA targets, respectively. In bacteria, *trans*-encoded sRNAs commonly require the assistance of the RNA chaperone Hfq to promote their interaction with the cognate mRNA targets. Although *cis*-encoded sRNAs share extended base pairing complementarity to their counterpart mRNAs, in a few cases, Hfq is required for their function (4). First identified in *Escherichia coli* as a host factor essential for the replication of the Qβ RNA phage, Hfq is now recognized as a global regulator of gene expression present in a wide variety of bacteria that impacts many molecular processes in bacterial physiology, stress response, and virulence (5, 6). The importance of the RNA-binding protein Hfq was uncovered by the characterization of *hfq* null mutants in diverse bacterial pathogens (7, 8). Further detailed research in its function in different bacteria showed that Hfq is a key posttranscriptional regulator, stabilizing sRNAs or facilitating sRNA/mRNA interactions that inhibit or enhance translation initiation. Furthermore, Hfq can act independently to modulate gene expression by affecting mRNA translation (for reviews, see references 6 and 9). Although deep sequencing approaches have revealed a high number and broad spectrum of sRNAs in diverse pathogens, such as *Salmonella enterica* serotype Typhimurium (10), *Pseudomonas aeruginosa* (11), *Yersinia pseudotuberculosis* (12), or *Legionella pneumophila* (13), the extent of Hfq-mediated riboregulation is highly complex and variable for each RNA type and in each organism. Furthermore, Hfq-associated sRNAs have been reported to control gene expression of multiple targets, thus regulating diverse cellular pathways, such as biofilm formation (14), catabolite repression (15), quorum sensing (16), or the control of transcriptional factors (17). Hfq is closely related to the Sm family of RNA-binding proteins in archaea and eukaryotes and phylogenetically widespread among bacteria, as about half of the sequenced bacterial genomes harbor at least one copy of the *hfq* gene (4, 18).

*Legionella pneumophila* is an intracellular bacterium that inhabits environmental aquatic systems, like lakes and rivers where it replicates in aquatic protozoa, but it can also infect humans to cause a severe pneumonia, and it also carries a gene that encodes Hfq (19, 20). However, little is known about the role of Hfq in the *L. pneumophila* life cycle or its regulatory function. The change between extra- and intracellular life and between replication in a host (replicative phase) and transmission to a new host (transmissive/virulent phase) demands a highly fine-tuned regulatory network (21). Indeed, the life cycle switch from replicating to transmissive/virulent *L. pneumophila* is governed through the function of several key regulators. Probably the most important ones are the two-component system (TCS) LetA/LetS (*Legionella* transmission activator and sensor, respectively) that induces traits necessary for efficient host transmission (22–24) and CsrA (carbon storage regulator) that is a posttranscriptional regulator, repressing transmissive/virulence traits during replication of *L. pneumophila* and releasing them in later stages of infection (25, 26; T. Sahr, C. Rusniok, F. Impenes, G. Oliva, O. Sismeiro, J. Y. Coppee, and C. Buchrieser, unpublished data). Moreover, the three sRNAs RsmX, RsmY, and RsmZ that are sequestering CsrA in transmissive phase to allow virulence traits to be translated are indispensable in this regulatory cascade (27, 28).

Here we report that *L. pneumophila* Hfq is regulated in a life cycle-dependent manner by a unique sRNA, named Anti-hfq that is transcribed in the early phase of the *L. pneumophila* life cycle. Our data support a complex model of regulation of the *hfq* transcript by the Anti-hfq sRNA, in which the Hfq chaperone together with RNase III are engaged to ensure the growth phase-dependent expression of this RNA-binding protein. Moreover, our results show that Hfq affects intracellular multiplication in amoebae, and consequently *L. pneumophila* virulence.

## RESULTS

### Hfq is highly conserved within the genus *Legionella* and other bacterial species.

**FIG 1:**
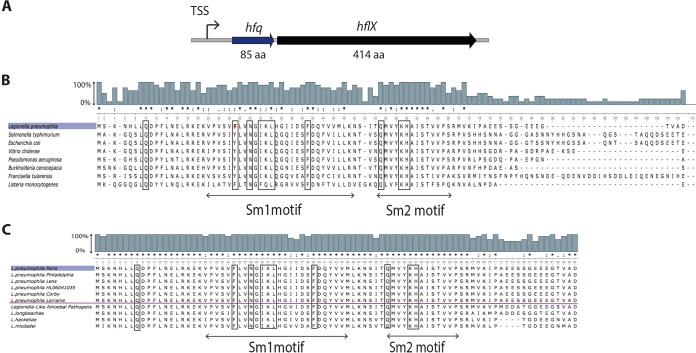
*Legionella* Hfq is conserved across the genus and other bacterial species. (A) Schematic organization of the *L. pneumophila hfq* locus. TSS, transcription start site; aa, amino acids. (B) Alignment of the *L. pneumophila* Hfq protein sequence with other bacterial Hfq protein sequences reveals high sequence and RNA binding site conservation. (C) Alignment of the *L. pneumophila* Paris Hfq protein sequence with the Hfq protein sequences from different *L. pneumophila* strains and other *Legionella or Legionella*-like species. Amino acids involved in RNA binding are boxed. Conserved amino acid residues (asterisks) and semiconservative substitutions (dots) and conservative substitutions (colons) are indicated. The bars above the sequence alignment indicate the sequence percentage of sequence conservation.

In *L. pneumophila*, Hfq is an 85-amino-acid protein encoded by the gene *lpp0009*. The *hfq* gene is organized in an operon with the putative GTP-binding protein HflX encoded by gene *lpp0010* (Fig. 1A). Although the *L. pneumophila hfq* gene shares the conserved chromosomal gene arrangement typical of other organisms like *E. coli* or *Vibrio cholerae* only partly, it shows high nucleotide and amino acid identity with Hfq of many Gram-negative bacteria (up to 70%) and Gram-positive bacteria (up to 50%). Furthermore, all residues that contribute to RNA binding are conserved in *L. pneumophila* (Fig. 1B). Comparison of the Hfq amino acid sequence among more than 300 *L. pneumophila* strains sequenced in the last years (19, 29–32) revealed that Hfq is 100% conserved across the different *L. pneumophila* strains. Analyses of four non-*pneumophila Legionella* species (33) showed that Hfq is 80% conserved (Fig. 1C).

### Hfq is highly expressed during postexponential/transmissive growth phase.

**FIG 2:**
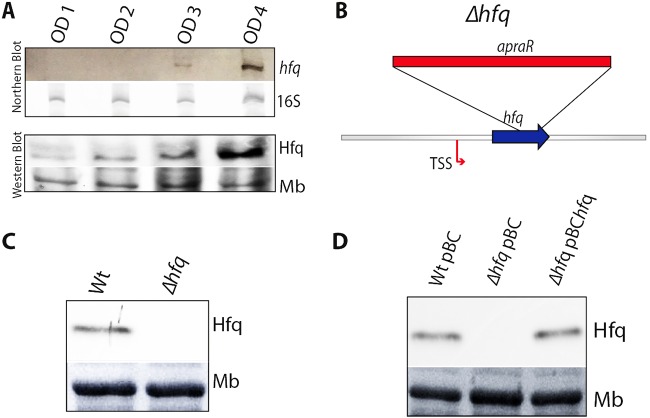
Transcript and protein expression of *hfq* are growth phase dependently regulated. (A) Northern blot and Western blot analyses of bacterial lysates from wild-type *L. pneumophila* Paris strain during growth (OD_600_s of 1, 2, 3, and 4) using an *hfq* probe and anti-Hfq antiserum, respectively. 16S RNA and the stained membrane (Mb) signals are shown as loading controls. (B) Schematic representation of the insertion of the apramycin resistance cassette (*apraR*) in the *Δhfq* mutant. (C) Detection of Hfq by Western blotting in the wild-type (wt) and *Δhfq* mutant strains grown to an OD_600_ of 4. (D) Detection of Hfq by Western blotting in the wild-type, *Δhfq* mutant, and complemented strain *Δhfq *pBC*hfq* (Wt and *Δhfq* carrying the empty plasmid pBC-KS) grown to an OD_600_ of 4.

In several pathogens, the level of expression of Hfq is growth phase dependent. In order to assess the transcriptional and posttranscriptional level of Hfq at different growth phases, we performed Northern and Western blot analyses of total RNA and whole protein lysates obtained from cultures of *L. pneumophila* (wild type [wt]) grown in liquid medium at 37°C. Northern blots using an *hfq*-specific probe showed very low *hfq* transcripts during exponential growth (optical density at 600 nm [OD_600_] of 2), but high transcript levels upon entry into postexponential growth (OD_600_ of 4). Protein expression followed the same pattern as shown by immunoblotting using anti-Hfq antibodies (Fig. 2A).

### Hfq is necessary for efficient intracellular replication at environmental temperatures.

In order to analyze the role and regulation of Hfq of *L. pneumophila*, we constructed an *hfq* deletion mutant (*Δhfq*) by the insertion of an in-frame apramycin resistance cassette (Fig. 2B). The resistance cassette used does not contain a transcriptional terminator; thus, transcription of the downstream gene, *hflX*, was not negatively affected as verified by transcriptome analyses (described below). Furthermore, the *Δhfq* mutant strain was completely sequenced using the Illumina technique, which ascertained that no secondary mutations had been introduced during the mutant construction. Analyses of the *Δhfq* mutant confirmed that the expression of Hfq was indeed abolished (Fig. 2C). To complement the *Δhfq* mutant, a plasmid harboring the entire *hfq* gene and its own promoter was transformed into the *Δhfq* mutant, generating the complemented strain *Δhfq* pBC*hfq*. Western blot analyses using anti-Hfq antibodies confirmed the expression of Hfq in *Δhfq* pBC*hfq* (Fig. 2D). In contrast to a previous report where the *Δhfq* mutant in another *L. pneumophila* strain showed a prolonged lag phase (20), the growth pattern of the *Δhfq* mutant analyzed here was very similar to that of the wt strain at 37°C (see Fig. S1A in the supplemental material) and at 20°C (Fig. S1B), indicating that the growth defect of a strain lacking Hfq is due to the intracellular environment in amoeba, and not to a general growth defect at lower temperatures.

10.1128/mBio.02182-16.1 FIG S1  *In vitro* growth of the *Δhfq* mutant at 37°C and 20°C is similar to that of the wild-type strain. (A) Growth of the *Δhfq* deletion mutant at 37°C. (B) Growth of the *Δhfq* deletion mutant at 20°C. Optical density values at 600 nm (OD_600_) of triplicate cultures in BYE medium were determined for 24 h (A) and for 96 h (B) (black, wild-type; red, *Δhfq*.) Download FIG S1, TIF file, 3.2 MB. Copyright © 2017 Oliva et al. 2017 Oliva et al. This content is distributed under the terms of the Creative Commons Attribution 4.0 International license.

**FIG 3:**
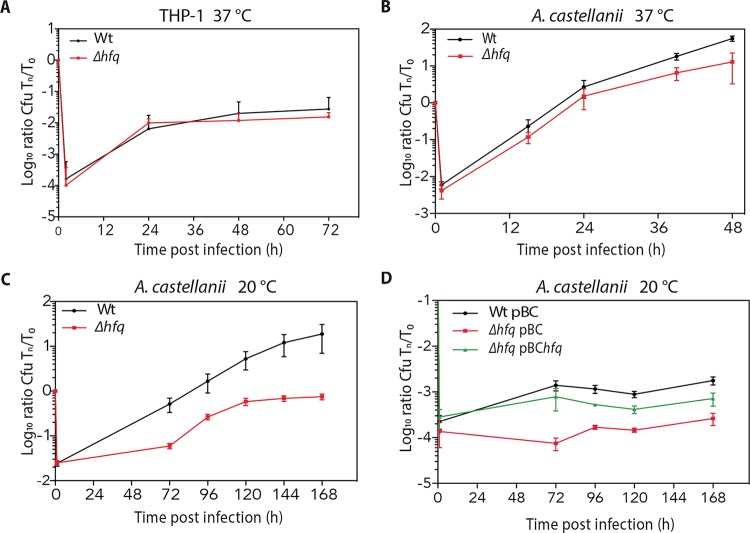
Efficient intracellular replication of *L. pneumophila* in *A. castellanii* and THP-1 macrophages is dependent on functional Hfq. (A) THP-1 cells were infected with wt and *Δhfq* mutant strains at an MOI of 10 at 37°C. The number of intracellular bacteria was monitored for 72 h, revealing a slightly diminished replication of the *Δhfq* mutant compared to the wt. (B and C) Monolayers of *A. castellanii* were infected with wt and *Δhfq* strains at an MOI of 0.1 at 37°C (B) and at an MOI of 1 at 20°C (C), showing a slight growth defect of the *Δhfq* mutant at 37°C but a clear defect at 20°C. (D) Infection of *A. castellanii* with the complemented *Δhfq* pBC*hfq* strain at an MOI of 1 at 20°C, showing complementation of the growth phenotype. The wt strain carrying plasmid pBC-KS, the *Δhfq* strain carrying the empty plasmid, and complemented strain *Δhfq* pBC*hfq* were examined. The number of intracellular bacteria was determined by recording the number of CFU per milliliter. Results are expressed at log_10_ ratio of CFU at *T*_*n*_/*T*0. Each time point represents the mean ± standard deviation (SD) (error bar) from at least three independent experiments.

To learn whether Hfq is implicated in virulence of *L. pneumophila* as reported for other bacterial pathogens, we compared the ability of the wt *L. pneumophila* and *Δhfq* mutant to infect and multiply in *Acanthamoeba castellanii* and in the human monocyte-derived cell line THP-1. Similarly to what was reported previously, the *Δhfq* mutant strain showed only a minimal growth defect in *A. castellanii* and THP-1 cells at 37°C (Fig. 3A and B). In contrast, when replication in *A. castellanii* was monitored at 20°C, the *Δhfq* mutant showed a clear replication defect compared to the wt strain (Fig. 3C). Furthermore, complementation of the *Δhfq* mutant restored the intracellular replication pattern (Fig. 3D). Taken together, our data imply that Hfq plays a role in intracellular replication in amoeba at environmental temperatures and thus on the virulence of *L. pneumophila*.

### Hfq expression is affected by RpoS and LetA.

**FIG 4:**
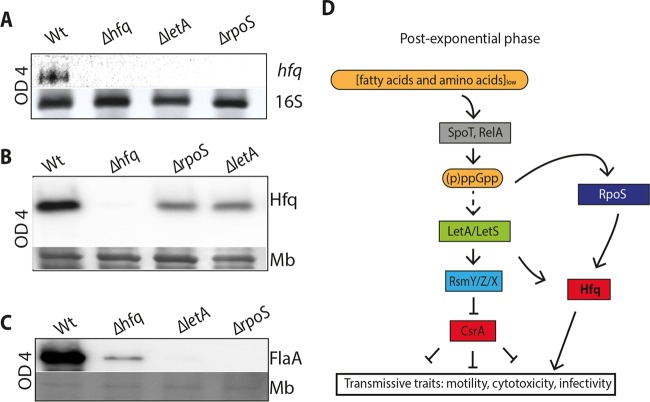
*hfq* transcript and protein expression are influenced by LetA and RpoS and impact flagellar expression. (A) Northern blot analyses of *hfq* transcripts in wt *L. pneumophila* and the *Δhfq*, *ΔletA*, and *ΔrpoS* regulatory mutants grown until they reached an OD_600_ of 4 show that the *hfq* transcript is under the control of LetA and RpoS and is abolished in the *Δhfq* mutant. (B) Western blot analysis of Hfq protein levels in wt *L. pneumophila* and *Δhfq*, *ΔletA*, and *ΔrpoS* mutants grown until an OD_600_ of 4 revealed a significantly decreased expression of Hfq in the regulatory mutants, indicating that RpoS and LetA influence Hfq expression. (C) Western blot analysis of FlaA protein levels in wt *L. pneumophila* and *Δhfq*, *ΔletA*, and *ΔrpoS* mutants grown until an OD_600_ of 4 revealed that expression of FlaA is strongly decreased in the *Δhfq* mutant and as expected missing in the *ΔletA* and *ΔrpoS* mutants, suggesting that Hfq also influences flagellar expression. Mb, stained membrane signal as a loading control. (D) Schematic overview of the major regulatory elements governing *L. pneumophila* virulence expression in transmissive/postexponential phase and the place and role of Hfq in this network.

The activation of virulence traits of *L. pneumophila* is highly regulated at the transcriptional and posttranscriptional levels. Major regulators implicated are the sigma factor RpoS and the two-component system LetA/LetS (21) (Fig. 4D). Hfq is another candidate, as the mutant showed a replication defect (Fig. 3C). To determine the role and place of Hfq in this regulatory network, we analyzed the *hfq* transcript and protein levels in *rpoS* and *letA* mutants. Northern blot analysis showed that *hfq* transcripts were abolished in *ΔrpoS* and *ΔletA* mutants, confirming that RpoS and LetA are implicated in the regulation of *hfq* expression (Fig. 4A). This was also reflected in the protein level, as observed by immunoblot analysis, where Hfq expression in the *ΔrpoS* and *ΔletA* mutants was strongly decreased compared to the Hfq levels in the wt strain (Fig. 4B). Thus, RpoS and LetA are strongly implicated in the regulation of Hfq expression at the transcript and protein levels. Flagella and consequently motility are hallmarks of the transmissive/virulent phase of *L. pneumophila*. We thus analyzed FlaA expression in the *Δhfq* mutant strain and the *ΔrpoS* and *ΔletA* mutants in which FlaA expression is known to be reduced. As expected, FlaA was highly expressed in the late postexponential phase in the wt but strongly reduced in the *Δhfq* mutant strain, suggesting its involvement in the regulatory cascade governing *L. pneumophila* differentiation, motility, and virulence (Fig. 4C). Taken together, the expression of Hfq in *L. pneumophila* is regulated in a growth phase-dependent manner and is influenced by RpoS and LetA. Furthermore, Hfq itself seems to be implicated in the activation of traits typical of the transmissive/virulent phase of *L. pneumophila*.

### Transcriptome analyses of the *Δhfq* mutant strain reveal only few changes in gene expression.

To analyze which genes Hfq is affecting that may lead to the decreased intracellular replication, transcriptome analysis at postexponential growth (OD_600_ of 4 grown *in vitro* in BYE medium and *in vivo* after 96 h of infection of *A. castellanii*) when Hfq is expressed the highest was performed. The comparison of the wt and *Δhfq* mutant transcriptomes *in vitro* identified only 18 differentially expressed genes (Table S1). This is in accordance with an *in vitro* transcriptome analysis of an *hfq* mutant in strain *L. pneumophila* JR32, where only a few genes and a mobile genetic element that excised upon the deletion of *hfq* were differentially expressed (34). *In vivo*, 74 genes were differentially transcribed due to the loss of Hfq, the majority of which (69 genes) was upregulated in the absence of Hfq, whereas only five genes were downregulated (Table S2). Interestingly, CsrA (0.43×), a major regulator of metabolic and regulatory functions during replication (Sahr et al., unpublished) was downregulated *in vivo*. In contrast, no effect of Hfq on other important regulators like RpoS or the two-component system LetA/LetS was seen on the transcript level, indicating no direct feedback cascade for this regulatory pathway. In total, eight genes were upregulated both *in vitro* and *in vivo*. Two of these genes are involved in flagellar assembly and motility (*flgG* and *flgH*), and two are coding for the enhanced entry protein EnhA (*lpp2693*) and EnhB (*lpp2694*), which are implicated in host cell infection (35). Additionally, the macrophage infectivity potentiator Mip, at least four Dot/Icm effector proteins, transcriptional regulators Fis1 and Fis2, and the DNA-binding protein HU-beta are differentially transcribed in the Δ*hfq* mutant during *in vivo* growth. These data might suggest a direct influence of Hfq on virulence formation as seen in infection of *A. castellanii*.

10.1128/mBio.02182-16.3 TABLE S1  Differentially expressed genes in *in vitro* analyses of wt and *Δhfq* strains grown in BYE broth at 37°C until OD_600_ of 4 (PE phase). Download TABLE S1, DOCX file, 0.1 MB. Copyright © 2017 Oliva et al. 2017 Oliva et al. This content is distributed under the terms of the Creative Commons Attribution 4.0 International license.

10.1128/mBio.02182-16.4 TABLE S2  Differentially expressed genes according to transcriptome *in vivo* analyses of wt and *Δhfq* strains grown for 96 h at 20°C within *A. castellanii*. Download TABLE S2, DOCX file, 0.1 MB. Copyright © 2017 Oliva et al. 2017 Oliva et al. This content is distributed under the terms of the Creative Commons Attribution 4.0 International license.

### An antisense RNA is present in the 5′ untranslated region of *hfq.*

**FIG 5:**
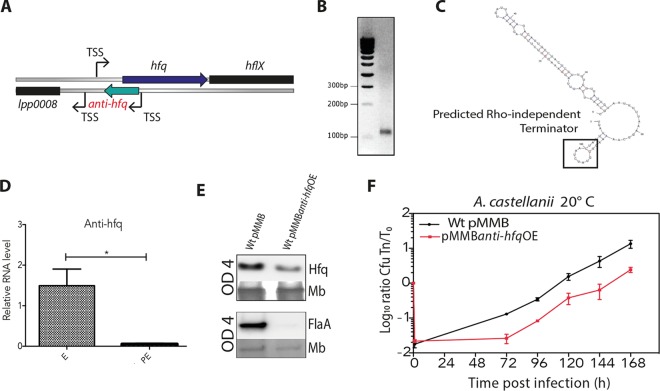
A small noncoding RNA named Anti-hfq is expressed antisense to *hfq* and influences Hfq expression and intracellular replication. (A) Schematic organization of the chromosomal organization of the *L. pneumophila hfq* and anti*-hfq* locus. (B) 3′ RACE PCR product in a 2% agarose gel obtained from exponentially grown wt *L. pneumophila* confirms the presence of an sRNA of 101 bp, named Anti-hfq. (C) Structure of the Anti-hfq sRNA of *L. pneumophila* as predicted by the program FindTerm. (D) qPCR analyses of the expression of Anti-hfq in the wt strain grown to exponential (E) phase and to postexponential (PE) phase, showing that Anti-hfq is expressed about 1.5 times in the E phase and 0.05 in the PE phase normalized to an OD_600_ of 1. *gyrB* and *tldD* were used as internal controls for normalization. Each time point represents the mean plus standard deviation from three independent experiments. The means for the wt strain at the E and PE phases were statistically significantly different (*P* < 0.05) by the *t* test as indicated by the bar and asterisk. (E) The anti*-hfq* sRNA influences Hfq and FlaA protein expression as evaluated by Western blotting analysis using the anti-Hfq or anti-FlaA antisera and lysates of wt and Anti-hfq-overexpressing (pMMB*anti-hfq*OE) strains grown to an OD_600_ of 4. Membrane (Mb) signals are shown as loading controls. (F) Infection of *A. castellanii* with the pMMB*anti-hfq*OE strain shows a similar growth defect as the *hfq* mutant strain, indicating a role in intracellular replication. Monolayers of *A. castellanii* were infected with wt and the pMMB*anti-hfq*OE strain at an MOI of 1 at 20°C. Intracellular replication was determined by recording the number of CFU per milliliter. Results are expressed in log_10_ ratio CFU *T*_*n*_/*T*0. Each time point represents the mean ± SD from three independent experiments.

We had previously established a complete transcriptional map of the *L. pneumophila* genome that revealed the presence of a dynamic pool of sRNAs regulated in a growth phase-dependent manner (13). Among these sRNAs, we identified a transcriptional start site (TSS) of a noncoding gene located in the reverse strand of the 5′ untranslated region (5′ UTR) of the *hfq* gene (Fig. 5A). In order to confirm experimentally the presence of a sRNA, we performed 3′ rapid amplification of cDNA ends (RACE), which yielded only a single band around 100 bp from RNA samples isolated from a culture grown at the early exponential phase (OD_600_ of 2). Cloning and sequencing of this cDNA amplimer that we named Anti-hfq showed that the noncoding RNA is 101 bp long (Fig. 5B and Fig. S2A). Using the program Mfold (36), the anti-*hfq* secondary structure was predicted to be composed of a duplex, with a 5′ overhang of 1 nucleotide (5′ C) and a 3′ overhang of 3 nucleotides (3′ UUA) containing a putative Rho-independent terminator identified by FindTerm (Softberry) (Fig. 5C). Although other programs did not confirm this terminator structure, the RACE PCR results showed that the transcript terminated at 101 bp where FindTerm predicted the terminator; hence, under the given conditions, Anti-hfq is indeed an sRNA. Bioinformatic analysis revealed the presence of an identical anti-*hfq* sequence among all *L. pneumophila* strains investigated. Anti-hfq homologues were also found among other *Legionella* species with a sequence identity of at least 80%, but no homologous sequences were found in other bacterial genomes. Thus, Anti-hfq represents a unique sRNA element within the genus *Legionella*.

10.1128/mBio.02182-16.2 FIG S2  Sequence of anti*-hfq* and control EMSA showing that no ternary complex is formed when an unrelated RNA is used. (A) Blue letters indicate the sequence of the small RNA; grey letters show the promoter region. The arrow indicates the flanking *lpp0008* starting codon. (B) EMSA using 25 nM radioactively labeled Anti-hfqHfq start codon together with 0, 10, 15, 30, or 50 nM cold *hfq* full transcript or 0, 15, 30, or 50 nM of the cold *lpp0644* RNA probes. The amount of cold RNA probe is indicated by the height of the black triangle above the lane. Abbreviations and symbols: (A), radioactively labeled Anti-hfq RNA probe; (h), radioactively labeled *hfq* mRNA probe; *, radioactively labeled RNA probes; −, the absence of the RNA probes. Download FIG S2, TIF file, 9.4 MB. Copyright © 2017 Oliva et al. 2017 Oliva et al. This content is distributed under the terms of the Creative Commons Attribution 4.0 International license.

### Anti-hfq is expressed at the early exponential phase of the *Legionella* growth cycle.

To determine the pattern of the Anti-hfq transcripts during the *L. pneumophila* life cycle, total RNA was extracted at exponential growth (OD_600_ of 1) and postexponential growth (OD_600_ of 4) of wt *L. pneumophila* grown in liquid BYE medium. The total RNA was reverse transcribed, and quantitative PCR (qPCR) analysis on the obtained cDNA was performed. We used different primer pairs: primer pair 1 (*hfq*-qPCR-F [F stands for forward] and *hfq*-qPCR-R [R stands for reverse]) exclusively recognizing the *hfq* mRNA and primer pair 2 (anti-*hfq*-qPCR-F and anti-*hfq*-qPCR-R) recognizing both the *hfq* and Anti-hfq RNAs, as these two transcripts entirely overlap (Fig. 5A). To confirm the growth phase-dependent expression of Anti-hfq, we calculated the ratio between the *hfq* and Anti-hfq transcript levels in the two growth phases. This showed that in the exponential phase, the Anti-hfq transcript was expressed about 1.5-fold higher than the *hfq* transcript, whereas its expression levels decreased to 0.05-fold compared to *hfq* in the postexponential phase (Fig. 5D). This alternative expression of either *hfq* or Anti-hfq suggests a regulation in which the expression of the Anti-hfq transcript might inhibit the expression of the sense transcript due to the *cis* regulatory function of the Anti-hfq sRNA.

### Anti-hfq affects intracellular replication.

To analyze whether the Anti-hfq sRNA indeed impacts Hfq expression levels, we first constructed a strain overexpressing Anti-hfq sRNA, in which the anti*-hfq* gene was cloned under the control of an isopropyl-β-d-thiogalactopyranoside (IPTG)-inducible promoter. Upon induction with IPTG, the overexpression of the Anti-hfq sRNA decreased Hfq expression levels compared to the wt (Fig. 5E, top blot), supporting the idea that Anti-hfq sRNA is able to directly regulate Hfq expression. As the deletion of *hfq* resulted in a strongly decreased flagellin expression (Fig. 4C), we postulated that the overexpression of the Anti-hfq sRNA should also impact flagellin expression via the repression of Hfq. Indeed, when the Anti-hfq sRNA was overexpressed, the expression of FlaA was strongly reduced compared to the wt strain (Fig. 5E, bottom blot), further suggesting a *cis* regulatory function of the Anti-hfq sRNA on Hfq expression. This result is consistent with a model in which an antisense sRNA regulates the transcription of its sense protein-coding gene, here *hfq*. The *L. pneumophila* Δ*hfq* mutant is attenuated in intracellular growth of *A. castellanii* (Fig. 3C), and flagellin is less well expressed in comparison to the wt strain (Fig. 4C). Thus, to test whether Anti-hfq has a role in intracellular replication of *L. pneumophila*, we infected *A. castellanii* with the strain overexpressing anti*-hfq*. At 72 h postinfection, 10-fold fewer intracellular bacteria were recovered from amoeba infected with the Anti-hfq sRNA-overexpressing strain (pMMB*antihfq*OE) compared to the wt, similar to the replication rate seen for the Δ*hfq* mutant strain (Fig. 5F and 3C). Thus, Anti-hfq sRNA plays a role in intracellular replication of *L. pneumophila*.

### Hfq expression is regulated by the Anti-hfq sRNA.

**FIG 6:**
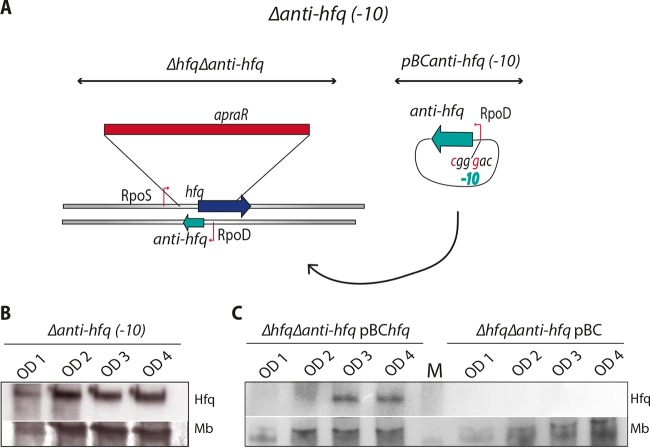
In an anti*-hfq* mutant, Hfq is already expressed during exponential growth. (A) Schematic presentation of the *Δhfq Δ*anti*-hfq* mutant and sequence changes introduced in the anti*-hfq* promoter region to construct the *Δ*anti*-hfq*(*-10*) mutant without disrupting the Hfq amino acid sequence. (B) The Anti-hfq sRNA influences Hfq protein expression as evaluated by Western blot analysis of Hfq in the *Δ*anti*-hfq*(*-10*) mutant strain. Stained membrane (Mb) signals are shown as a loading control. (C) Western blot analysis of Hfq protein levels in the *Δhfq Δ*anti*-hfq* mutant complemented with *hfq* and anti*-hfq* (*ΔhfqΔanti-hfq* pBC*hfq*) shows that the growth phase-dependent Hfq expression pattern is restored. In contrast, the control strain carrying the empty plasmid (*ΔhfqΔanti-hfq*pBC) does not express Hfq. Stained membrane (Mb) signals are shown as loading control. M, molecular weight marker.

In the *Δhfq* mutant used until now, the anti*-hfq* gene was still intact (Fig. 2B). Thus, to further study the function of Anti-hfq sRNA, we constructed a second mutant containing a larger deletion as the entire region spanning *hfq* and anti*-hfq* (*Δhfq Δ*anti*-hfq*) was replaced with an apramycin cassette (Fig. 6A). By complementing this mutant with the plasmid pBC*anti-hfq*(-*10*) in which two single mutations in the anti*-hfq* −*10* box had been introduced, we were able to study the role of the Anti-hfq sRNA without disturbing Hfq expression. This complemented strain was named the *Δ*anti*-hfq*(-*10*) strain (Fig. 6A). When analyzing the Hfq expression levels in the *Δ*anti*-hfq*(-*10*) mutant, the Hfq expression pattern differed compared to the wt strain, as the expression of the *hfq* transcripts started already during exponential growth of *L. pneumophila* (Fig. 2A, bottom blot, and Fig. 6B), indicating that Anti-hfq sRNA indeed represses *hfq* transcripts in exponential growth. In contrast, in the complemented mutant strain (Δ*hfq* Δanti*-hfq* pBC*hfq*), Hfq expression was restored to wt levels (Fig. 6C), whereas in the control strain (Δ*hfq* Δanti*-hfq* mutant carrying the empty plasmid; Δ*hfq* Δanti*-hfq* pBC), no expression of Hfq was seen, as expected (Fig. 6C). Thus, the antisense RNA Anti-hfq regulates Hfq expression levels in a growth phase-dependent manner by functioning as a *cis*-complementary sRNA.

### The *hfq* and Anti-hfq RNA transcripts interact *in vitro.*

**FIG 7:**
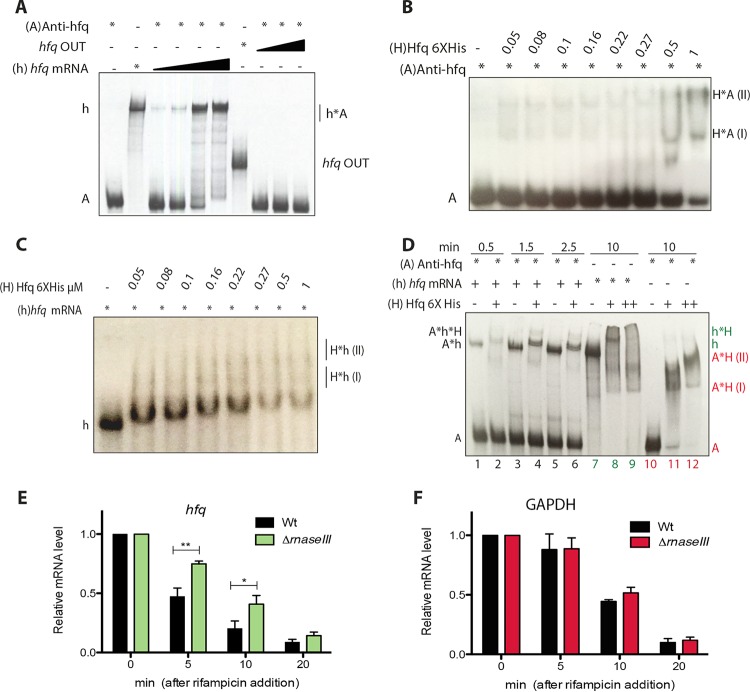
Anti-hfq regulates *hfq* expression through binding to its complementary region which is facilitated by Hfq. (A) EMSA using 25 nM radioactively labeled Anti-hfq and 0, 10, 15, 30, or 50 nM cold full *hfq* transcript or 0, 15, 30, or 50 nM *hfq*OUT as control RNA probes shows that Anti-hfq binds *hfq* mRNA. The amount of RNA probe is indicated by the height of the black triangle above the lane. (B and C) EMSAs using 25 nM radioactively labeled Anti-hfq (B) and *hfq* (C) RNA alone or with the indicated increasing molar amounts of Hfq protein, revealing that Anti-hfq and *hfq* bind Hfq. (D) A radioactively labeled Anti-hfq RNA probe and the cold *hfq* mRNA probe were incubated (lanes 1, 3, and 5), showing the formation of a duplex complex or with 1 μM Hfq protein (lanes 2, 4, and 6) showing the formation of a ternary complex. The ability of the protein to bind separately was evaluated by incubating radioactively labeled *hfq* (lanes 7 to 9) or Anti-hfq (lanes 10 to 12) RNA probes for 10 min. The duplex and ternary complexes were incubated for 0.5 min (lanes 1 and 2), 1.5 min (lanes 3 and 4), and 2.5 min (lanes 5 and 6) at room temperature. The resulting complexes were analyzed on an 8% native polyacrylamide gel as described in Materials and Methods. Abbreviations: (A), radioactively labeled Anti-hfq RNA probe; (h), radioactively labeled *hfq* mRNA probe; (H), Hfq6XHis; (I) and (II), formation of complexes. Symbols: *, radioactively labeled RNA probes; +, cold RNA probes; −, no RNA probes. (E and F) RNA stability assays reveal the RNase III dependence of the *hfq* transcript mRNA *in vivo*. Wt and RNase III deletion strains were grown in BYE medium before rifampin treatment, showing that RNase III-dependent *hfq* mRNA decay was favored. The graphs show the relative amount of *hfq* (E) and GAPDH (F) mRNA remaining at each time point in the wt and RNase III gene deletion strains. 16S was used as internal control for normalization. Each time point represents the mean plus standard deviation from three independent experiments. The quantitative data were analyzed using two-way analysis of variance (ANOVA) test with Bonferroni posttest. A *P* of <0.05 was considered to be statistically significant. The values that are significantly different are indicated by a bar and asterisk as follows: **, *P* < 0.01; *, *P* < 0.05.

Our previous results suggest a regulation of the *hfq* transcript through binding of its Anti-hfq antisense sRNA. To investigate a direct interaction of Anti-hfq and *hfq* mRNA *in vitro*, we performed electrophoretic mobility shift assays (EMSAs). Incubation with a radioactively labeled Anti-hfq RNA probe and increasing concentrations of cold *hfq* mRNA resulted in a slower-migrating complex, suggesting a direct interaction of the two RNA molecules (Fig. 7A). In contrast, when the EMSA was performed with Anti-hfq sRNA and a truncated *hfq* mRNA probe spanning the nucleotides 78 to 255 missing the 5′ UTR region and the first 26 codons (*hfq* OUT), no changes in terms of migration were observed, consistent with the absence of formation of a complex (Fig. 7A). Similar results were obtained when using the mRNA of an unrelated gene (*lpp0644* RNA probe) as a second negative control (Fig. S2B). Thus, Anti-hfq forms an RNA duplex with the *hfq* mRNA and most likely regulates *hfq* mRNA expression by direct binding due to complementarity.

### Purified Hfq binds *hfq* and Anti-hfq sRNA with different affinity.

Although the Hfq protein is known to facilitate the interaction between *trans*-encoded sRNAs and their mRNA targets, the Hfq chaperone may also function to stabilize/destabilize *cis*-encoded sRNAs and their complementary mRNA targets. Thus, we sought to determine whether the Hfq protein might be able to form complexes either with the *hfq* mRNA or with the Anti-hfq sRNA. The analysis of the *hfq* and anti*-hfq* sequences revealed the presence of (AAN)_*n*_ triplets and AU-rich regions, which could be Hfq binding regions, further suggesting the hypothesis of an Hfq autoregulatory loop. To assess the ability of Hfq to bind *hfq* and Anti-hfq transcripts separately, we evaluated binding *in vitro* by EMSAs using recombinant Hfq protein. As shown in Fig. 7B and C, Hfq interacts with both RNA molecules but with different affinities.

To study the inhibitor complex formed by the *hfq* mRNA, Anti-hfq, and the Hfq protein in more details, we employed a gel-shift kinetic assay (Fig. 7D). A radioactively labeled Anti-hfq RNA probe was incubated with 25 nM of cold *hfq* mRNA in the absence (Fig. 7D, lanes 1, 3, and 5) or presence (Fig. 7D, lanes 2, 4, and 6) of Hfq protein for 0.5 (lanes 1 and 2), 1.5 (lanes 3 and 4), and 2.5 (lanes 5 and 6) minutes. As shown above, the two RNA molecules were able to interact. Additionally, we detected a strong band corresponding to the formation of a ternary complex already after 0.5 min of incubation. Moreover, the intensities of the shifted bands indicated that the affinity of Hfq for the RNA-RNA complex might be much stronger than for the single RNAs alone. The super shift and thus the formation of the ternary complex was increasing with longer incubation time (after 1.5 and 2.5 minutes). To test the specificity of this complex, radioactively labeled *hfq* or Anti-hfq probes were incubated alone in parallel with increasing amounts of Hfq confirming that Hfq is indeed able to bind each of the RNA molecules separately (Fig. 7D, lanes 7 to 9 and 10 to 12). Therefore, although Anti-hfq is complementary to its own target and thus it should not require Hfq for binding, Hfq is able to bind the two RNA molecules, forming a ternary complex.

### RNase III might participate in the double-strand RNA (dsRNA) regulation.

One of the regulatory functions of the Hfq RNA chaperone is the recruitment of RNases for the degradation of sRNA and/or mRNA targets. Thus, we wondered whether RNases might be in involved in the degradation of the ternary complex in *L. pneumophila*. To answer this question, we performed an RNA stability assay in the wt and an RNase III gene (*lpp1834*) deletion mutant that we constructed. Analysis of the *hfq* mRNA levels, after the addition of rifampin, showed a half-life of 4.1 min in the wt strain and of 8.2 min in the RNase III deletion mutant (Fig. 7E). In contrast, when the half-life of the glyceraldehyde-3-phosphate dehydrogenase (GAPDH) transcript was determined in the same conditions, no significant differences were observed in the relative mRNA levels between the wt and the RNase III deletion mutant (Fig. 7F). This strongly suggests that RNase III is involved in the cleavage of the *hfq*–Anti-hfq RNA duplex and hence, affects the stability of the *hfq* mRNA, closing the Hfq regulation loop.

## DISCUSSION

*Legionella pneumophila* needs to adapt to many different environmental conditions, including low-temperature and nutrient-poor aquatic and hostile intracellular environments of protozoa or human macrophages. To regulate the transition from one environment to another environment, *L. pneumophila* has evolved a complex regulatory cascade allowing it to switch from a replicative stage to a transmissive/virulent stage (21). This regulatory network is comprised of many global regulators like the RNA-binding protein CsrA and its small noncoding RNAs RsmX, RsmY, and RsmZ, the TCS LetA/LetS, and the stress sigma factor RpoS (22–28; Sahr et al, unpublished). Here we demonstrate that the RNA chaperone Hfq is another major player in the regulation of the switch to transmissive/virulent *L. pneumophila* and that life cycle-dependent Hfq expression is regulated by an antisense RNA named Anti-hfq.

Comparative sequence analyses showed that Hfq is highly conserved and present in all *L. pneumophila* strains sequenced thus far (Fig. 1B). Our observation that the *hfq* transcript and the Hfq protein are barely expressed at early stages of growth but highly expressed at the postexponential phase of growth (Fig. 2A) establishes Hfq as a growth phase-dependent regulated protein and suggests its implication in the regulation of the expression of virulence traits, a feature of postexponential bacteria. Interestingly, in 2005, McNealy and colleagues (20) had reported that Hfq of *L. pneumophila* JR32 is expressed in the exponential phase of growth and is positively regulated by the stationary-phase sigma factor RpoS. Furthermore, they proposed that upon entry into stationary phase, Hfq expression is abolished through the regulatory function of the two-component regulator LetA, thereby ensuring that *hfq* transcripts are off when the infectious traits need to be activated (20). The differences from our results might be due to the different strains used or perhaps to the excision of the 100-kb plasmid pL100 when *hfq* is deleted, as reported by Trigui and colleagues (34). However, our results are in agreement with the *hfq* expression pattern observed in several other bacterial pathogens such as *P. aeruginosa* and *Listeria monocytogenes* (37, 38) but also with the life cycle of *L. pneumophila* (21, 39). The regulation of virulence traits by Hfq, which demands its expression in the postexponential growth phase, is supported by the observation that the *hfq* mutant is defective in intracellular growth, a characteristic also reported by McNealy and colleagues, and the transcriptome results identifying virulence genes and virulence gene regulators to be differentially expressed upon deletion of *hfq* (see Table S1 and S2 in the supplemental material). By analyzing the protein and transcript levels of Hfq in different regulatory mutants, we show that Hfq expression is influenced by the stationary sigma factor RpoS and the response regulator LetA during the postexponential phase, as both directly or indirectly turn on *hfq* transcription (Fig. 4A and B). Thus, Hfq plays an important role in the regulatory cascade governing the switch to the transmissive phase of *L. pneumophila* (Fig. 4D).

In agreement with the position of Hfq in this regulatory network, the loss of Hfq impaired intracellular replication at 20°C, the optimal growth temperature of *A. castellanii* and a temperature that is close to environmental conditions (Fig. 3C). The transcriptome analysis of the Δ*hfq* mutant during infection of *A. castellani* supported this finding, as several secreted effector proteins, the enhanced entry proteins EnhABC, the global DNA-binding transcriptional regulators Fis1 and Fis2, and the DNA-binding protein HU-beta were differentially regulated in the *hfq* mutant. Moreover, the above-mentioned regulators are all related to environmental adaptation, virulence, and stress response regulation and fitness in different pathogenic bacteria (40). Furthermore, a hallmark of transmissive/virulent *L. pneumophila*, the expression of flagellar protein FlaA that is intimately linked to virulence, was strongly reduced in the *Δhfq* mutant at an OD_600_ of 4, similar to what is seen in LetA and RpoS mutants (Fig. 4C). Collectively, these results indicate that *L. pneumophila* requires Hfq to promote motility and to efficiently multiply within *A. castellanii* at environmental temperatures.

Most studies of Hfq analyzed its role in the regulation of sRNAs and their mRNA targets, but not how Hfq expression itself is regulated. *L. pneumophila* Hfq is clearly growth phase dependently regulated, as transcript and protein levels are low during replicative/exponential growth but are strongly expressed in transmissive/postexponential growth (Fig. 2A). This growth phase-dependent regulation is achieved by an sRNA that we named Anti-hfq as it is transcribed on the antisense strand of the *hfq* gene overlapping its 5′ UTR (Fig. 5A). Anti-hfq is a 101-bp long sRNA that is highly expressed during exponential growth, but its expression is strongly decreased upon entry into the transmissive/postexponential growth phase. These opposite expression patterns of the *hfq* and Anti-hfq transcripts together with the fact that the sRNA is encoded antisense to *hfq* suggested that it has a role in regulating *hfq* expression. Furthermore, the identification of a partly conserved LetA binding site (two mismatches) suggested that the growth phase-dependent expression of Anti-*hfq* sRNA might be regulated by LetA. However, we could not firmly establish a specific interaction; thus, this regulatory pathway remains to be analyzed in the future. A detailed analysis of the anti*-hfq* sequence revealed the presence of a putative Rho-independent transcriptional terminator as described in a large part of functional Hfq binding modules of sRNAs (41). Furthermore, the ARN or ARNN (R is purine, and N is any nucleotide) motifs that are preferentially bound in the distal site of the Hfq homohexamer (42) were also present in the proximity of the *hfq* ribosome binding site (RBS), and we showed that Anti-hfq sRNA binds the complementary region of the *hfq* mRNA. Furthermore, Hfq is able to interact separately with both RNA molecules, *hfq* and Anti-hfq (Fig. 7B and C), but it also forms a ternary complex, suggesting an autoregulatory circuit (Fig. 7D). Finally, the riboendonuclease RNase III takes part in the regulation of Hfq probably cleaving the double-strand RNA as suggested by RNA stability measurements in an RNase III mutant strain (Fig. 7E). Several studies of *E. coli* had suggested that Hfq binds two distinct sites of the 5′ UTR of its own mRNA, hindering the formation of the translation initiation complex and thus negatively regulating its own expression. In *E. coli*, RNase E is recruited to exert its RNase function to degrade *hfq* mRNA (43). Thus, collectively, the data suggest that binding of the *cis*-encoded Anti-hfq sRNA obstructs Hfq translation in exponential growth (Fig. 7A).

**FIG 8:**
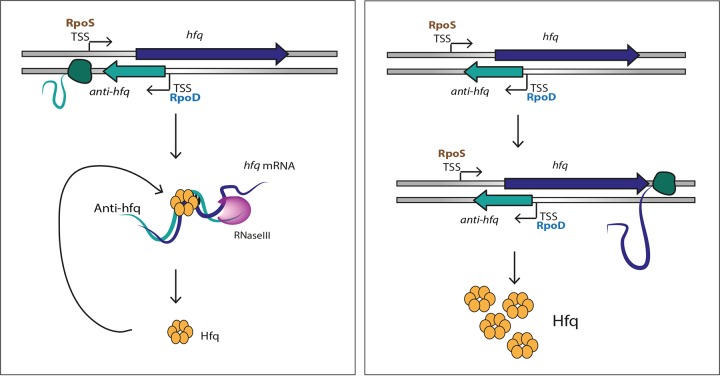
Model of the regulation of Hfq in replicative and transmissive *L. pneumophila*. During the replicative phase, the Anti-hfq sRNA is highly expressed and represses Hfq expression through binding to the *hfq* mRNA. This process also involves Hfq itself, which autoregulates its own expression and the riboendonuclease RNase III that likely cleaves the *hfq* mRNA product. In contrast, upon entry into the transmissive phase, Anti-hfq is not expressed, leading to high Hfq expression that now influences the expression of motility and virulence traits of *L. pneumophila*.

The regulation of Hfq by a *cis-*encoded sRNA is an unusual feature. We propose that binding of the *cis*-encoded Anti-hfq sRNA to *hfq* mRNA in exponential growth leads to low translation of Hfq, whereas when the expression of Anti-hfq sRNA decreases in the transmissive phase, high expression of Hfq is possible. This leads to the expression of several Dot/Icm secreted substrates, global regulators like Fis1 and Fis2 that are implicated in the regulation of virulence traits (44) and probably of several of the many growth phase dependently regulated sRNAs that we identified earlier (13) (Fig. 8). Thus, *L. pneumophila* is equipped with a highly sophisticated regulatory mechanism further fine-tuning the regulation of the reciprocal expression of distinct sets of genes under different environmental conditions.

## MATERIALS AND METHODS

### Bacterial strains, growth media, and culture conditions used.

The bacterial strains used in this study are listed in Table 1. *L. pneumophila* strain Paris and its derivatives were cultured in *N*-(2-acetamido)-2-aminoethanesulfonic acid (ACES)-buffered yeast extract broth (BYE) or on ACES-buffered charcoal-yeast (BCYE) extract agar (45), and *E. coli* was grown in Luria-Bertani (LB) broth and agar. All strains were grown at 37°C. For the construction of knockout mutants and complementation plasmids, antibiotics were used at the following concentrations: ampicillin at 100 mg/ml, kanamycin at 50 mg/ml, and chloramphenicol at 20 mg/ml for *E. coli*; and kanamycin at 10 mg/ml, chloramphenicol at 20 mg/ml, and apramycin at 15 mg/ml for *L. pneumophila*. *A. castellanii* ATCC 50739 was cultured in PYG 712 medium [2% proteose peptone, 0.1% yeast extract, 0.1 M glucose, 4 mM MgSO_4_, 0.4 M CaCl_2_, 0.1% sodium citrate dihydrate, 0.05 mM Fe(NH_4_)_2_(SO_4_)_2_ ⋅ 6H_2_O, 2.5 mM NaH_2_PO_3_, 2.5 mM K_2_HPO_3_] at 20°C. THP-1 human monocytes were grown in RPMI 1640 GlutaMAX medium (Gibco) supplemented with 10% fetal bovine serum at 37°C and 5% CO_2_.

**TABLE 1:** Bacterial strains and plasmids used in the study

Strain or plasmid	Description^a^	Reference or source
Strains		
*L. pneumophila* CIP 107629	*L. pneumophila* serogroup 1 strain Paris (*Lp*P)	19
*L. pneumophila* pBC	*Lp*P carrying pBC-KS	54
*L. pneumophila* pMMB207C	*Lp*P carrying pMMB207C	28
*L. pneumophila* Δ*hfq*	*Lp*P *hfq*::Apr^r^	This study
*L. pneumophila* Δ*hfq* pBC	*Lp*P *hfq*::Apr^r^ carrying pBC-KS	This study
*L. pneumophila* Δ*hfq* pBC*hfq*	*Lp*P *hfq*::Apr^r^ carrying pBC*hfq*	This study
*L. pneumophila* Δ*hfq* Δ*anti-hfq*	*Lp*P *hfq* anti*-hfq*::Apr^r^	This study
*L. pneumophila* Δ*hfq* Δ*anti-hfq* pBC	*Lp*P *hfq* anti*-hfq*::Apr^r^ carrying pBC*hfq*	This study
*L. pneumophila* Δ*hfq* Δ*anti-hfq* pBC*hfq*	*Lp*P *hfq* anti*-hfq*::Apr^r^ carrying pBC*hfq*	This study
*L. pneumophila* Δ*anti-hfq* (*-10*)	*Lp*P *hfq* anti*-hfq*::Apr^r^ carrying pBC*hfqanti-hfq*(*-10*)	This study
*L. pneumophila* pMMB*anti-hfq*OE	*Lp*P carrying pMMB*anti-hfq*OE	This study
*L. pneumophila* Δ*letA*	*Lp*P *letA*::Km^r^	28
*L. pneumophila* Δ*rpoS*	*Lp*P *rpoS*::Km^r^	13
*L. pneumophila* Δ*rnaseIII*	*Lp*P carrying the RNase III gene fused to the Apr^r ^cassette	This study
*E. coli* DH5α	F^−^ ϕ80d*lacZ*ΔM15 Δ(*lacZYA-argF*)*U169 deoR recA1 endA1 hsdR17*(r_K_^−^ m_K_^+^ *phoA supE44* λ^−^ *thi*-*1 gyrA96 relA1*	Invitrogen
Plasmids		
pGEM-T Easy	Cloning of PCR products; Amp^r^	Promega
pBC-KS	Expression vector; Cm^r^	Stratagene
pMMB207C	*Legionella* expression vector; Δ*mobA*; Cm^r^	55
pBC*hfq*	pBC-KS containing *hfq* and anti*-hfq* genes; Cm^r^	This study
pMMB*anti-hfq*OE	pMMB207C containing anti*-hfq* gene under the p*tac* promoter; Cm^r^	This study
pBC*anti-hfq*(-*10*)	pBC*hfq* mutated in the −10 upstream region of anti*-hfq*; Cm^r^	This study

a Abbreviations: Amp^r^, ampicillin resistance; Apr^r^, apramycin resistance; Cm^r^, chloramphenicol resistance; Km^r^, kanamycin resistance.

### Mutant and plasmid constructions.

The plasmids and oligonucleotide primers used in this study are listed in Table 1 and 2, respectively. Mutant strains of *L. pneumophila* were constructed as previously described (39, 46). In brief, the gene of interest was inactivated by introduction of an apramycin resistance (Apr^r^) cassette. The mutant alleles were constructed using a three-step PCR. For the construction of the *Δhfq* deletion mutant strain, three overlapping fragments (*lpp0009* upstream region primers *hfq*-Mut_F and *hfq-*apra_R, antibiotic cassette-primers apra_F and apra_R, *lpp0009* downstream region primers *hfq-*apra_F and *hfq*-Mut_R; Table 2) were amplified independently and purified on agarose gels. The three resulting PCR products were mixed at the same concentration (15 nM), and a second PCR with flanking primers (primers *hfq*-Mut_F and *hfq*-Mut_R) was performed. This PCR product, the resistance marker cassette flanked by 300-bp regions homologous to *lpp0009* was introduced into the *L. pneumophila* Paris strain by natural competence (47). Strains that had undergone allelic exchange were selected by plating on BCYE containing apramycin, and the mutant was verified by PCR and sequencing. For the construction of the *Δhfq Δ*anti*-hfq* double mutant strains and the RNase III mutant, the same cloning strategy was used, and the primers are listed in Table 2.

**TABLE 2:** Primers used in this study

Primer	Primer sequence (5′ −3′)	Purpose	Reference
*hfq*-Mut_F	AAGAATTGATCAGGCCTGTC	Deletion of the *hfq* gene	This study
*hfq*-Mut_R	CCGACGATGCGTAAATTGGA	Deletion of the *hfq* gene	This study
apra_F	TTCATGTGCAGCTCCATCAGC	Deletion of the *hfq* gene	This study
apra_R	GAGCGGATCGGGGATTGTCTT	Deletion of the *hfq* gene	This study
*hfq-*apra_R	GCTGATGGAGCTGCACATGAATGCAATTTAATACCATTGACCAGG	Deletion of the *hfq* gene	This study
*hfq-*apra_F	GAGCGGATCGGGGATTGTCTTTCTGGTGAGGAAGAAGGAACTG	Deletion of the *hfq* gene	This study
*hfqanti-hfq1_*F	ACACTCCAAAACGAGGCGGCTG	Deletion of the *hfq* and anti*-hfq* genes	This study
*hfqanti-hfq2*_R	GCTGATGGAGCTGCACATGAACGGGTATCTAACTATTTATTCGA	Deletion of the *hfq* and anti*-hfq* genes	This study
*hfqanti-hfq2*_F	GAGCGGATCGGGGATTGTCTTACTGTGGCAGACTAATCAATTTA	Deletion of the *hfq* and anti-*hfq* genes	This study
*hfqanti-hfq1_*R	CGACATCCAAATAATCGCTCG	Deletion of the *hfq* and anti-*hfq* genes	This study
Hfq_comple_F	AAGCTTGCCAGTCTCAATGCAATTGCG	Complementation of *hfq* and *hfqanti-hfq*	This study
Hfq_comple_R	GTCGACTTGATTAGTCTGCCACAGTTCC	Complementation of *hfq* and *hfqanti-hfq*	This study
M-10*anti-hfq_*F	ATTGACCAGGAACACTGAAACCGGGACCTTTTCCTTGCGCAATTCATT	Mutation of the −10 promoter of anti*-hfq*	This study
M-10*anti-hfq_*R	AATGAATTGCGCAAGGAAAAGGTCCCGGTTTCAGTGTTCCTGGTCAAT	Mutation of the −10 promoter of anti*-hfq*	This study
anti-*hfq*_OE_F	TCTAGAGCGCAATTCATTTAGGAAAGG	Overexpression of anti*-hfq*	This study
anti-*hfq*_OE_R	CTGCAGAAACCACGCTGTCATGAAAATATAC	Overexpression of anti*-hfq*	This study
anti-*hfq*_3′ RACE_F	TTTAGGAAAGGGTCTTGTAGTAAATG	3′ RACE anti*-hfq*	This study
anti-*hfq*_3′ RACE_R	AATAGTTAGATACCCGTTTTTGCC	3′ RACE anti*-hfq*	This study
*rnaseIII*_Mut_F	ATGCGCTCAGCAATTGAATTAGC	Deletion of the RNase III gene	This study
*rnaseIII*_Mut_R	TCTGGTCTGGATGAGTTGGAATG	Deletion of the RNase III gene	This study
*rnaseIII*_Inv_F	GAGCGGATCGGGGATTGTCTTTATCGCTACCAGCACTGCAATG	Deletion of the RNase III gene	This study
*rnaseIII*_Inv_R	GCTGATGGAGCTGCACATGAATGTAACATGCACAATTGAGGGAG	Deletion of the RNase III gene	This study
anti-*hfq* RNA_T7_F	TAATACGACTCACTATAGGCGCAATTCATTTAGGAAAGGG	*In vitro* transcription of anti*-hfq*	This study
anti-*hfq* RNA_T7_R	T AGTTAGATACCCGTTTTTGCC	*In vitro* transcription of anti*-hfq*	This study
*hfq*mRNA_T7_F	TAATACGACTCACTATAGGGATAGGGTGTCGAATAAATAG	*In vitro* transcription of *hfq* mRNA	This study
*hfq*mRNA_T7_R	TTGATTAGTCTGCCACAGTTCC	*In vitro* transcription of *hfq* mRNA	This study
*lpp0644* _T7_F	TAATACGACTCACTATAGGGGAATGTTATGAGTGACTTG	*In vitro* transcription of *lpp0644*	This study
*lpp0644*_T7_R	TCCAGTCGTCTGCGCGCATCC	*In vitro* transcription of *lpp0644*	This study
*hfqOUT*_T7_F	TAATACGACTCACTATAGGGTCAATGGTATTAAATTGCATGGG	*In vitro* transcription of *hfq* mRNA missing the 5′ UTR	This study
anti-*hfq*_qPCR_F	TTTAGGAAAGGGTCTTGTAGTAA	qPCR analysis of the anti-*hfq* region overlapping *hfq* mRNA	This study
anti-*hfq*_qPCR_R	AATAGTTAGATACCCGTTTTTGCC	qPCR analysis of the anti-*hfq* region overlapping *hfq* mRNA	This study
*tldD*_qPCR_F	AATCGGAACGTCGATGATGCTG	qPCR analysis of the *tldD* mRNA	This study
*tldD*_qPCR_R	ATCCCTACCCCCTTATCCAGAG	qPCR analysis of the *tldD* mRNA	This study
*gyrB*_qPCR_F	GAGCGTAGACGCCAGTTATGA	qPCR analysis of the *gyrB* mRNA	This study
*gyrB_*qPCR_R	TGATGCAAACCGGTTCCATCA	qPCR analysis of the *gyrB* mRNA	This study
*hfq*mRNA_NB_F	TAATACGACTCACTATAGGGAACAACTGTTGAAATGGCGTG	Northern blot analysis of *hfq* mRNA	This study
*hfq*mRNA_NB_R	GTTTCAGTGTTCCTGGTCAATGG	Northern blot analysis of *hfq* mRNA	This study
*hfq_*qPCR_F	TCAGTGTTCCTGGTCAATGG	Determination of *hfq* mRNA half-life	This study
*hfq_*qPCR_R	AACAACTGTTGAAATGGCGTG	Determination of *hfq* mRNA half-life	This study
*gapdH*_qPCR_F	TTGATACGACAGTGGTCTATGG	Determination of GAPDH mRNA half-life	This study
*gapdH*_qPCR_R	CATGGACAGTGTTGACTAAGCC	Determination of GAPDH RNA half-life	This study
16S_qPCR_F	TTGTCTAGCTTGCTAGACAGATGG	Determination of 16S half-life	This study
16S_qPCR_R	AGCTTTCGTCCTCAGACATTATGC	Determination of 16S half-life	This study

For complementation experiments, the region, including *lpp0009* and *lnc0003* was PCR amplified with primers containing HindIII and SalI restriction sites at their ends (Hfq_compl_F and Hfq_compl_R) and ligated to the pBC-KS plasmid, previously digested with the two restriction enzymes. The resulting plasmid, named pBC*hfq*, was introduced into the *Δhfq* and *Δhfq Δ*anti*-hfq* deletion mutant strains by electroporation. The wild-type (wt) *L. pneumophila* Paris, the *Δhfq* and the *Δhfq* Δanti*-hfq* deletion mutant strains containing the empty plasmid pBC-KS were used as control. For constructing the Anti-hfq mutant strain, site-directed mutagenesis of anti*-hfq* was performed on the pBC*hfq* plasmid as the template using the QuikChange site-directed mutagenesis kit (Qiagen) following the manufacturer’s instructions. Two mutations were introduced in the −10 promoter region of the anti*-hfq* gene using the primers M-10anti*-hfq_*F and M-10anti-*hfq*_R. The resulting plasmid, pBC*anti-hfq*(-*10*), was introduced into the *Δhfq Δ*anti*-hfq* deletion mutant, creating the *Δ*anti*-hfq*(*-10*) mutant strain.

For overexpression of Anti-hfq sRNA in *L. pneumophila*, we used the pMMB207C (derived from pTS-10, kindly provided by H. Hilbi [48]). The anti*-hfq* gene was amplified using primers containing XbaI and PstI restriction sites (*anti-hfq*_OE_F and *anti-hfq*_OE_R primers) and ligated into pMMB207C, linearized using the same restriction enzymes. The resulting plasmid (pMMB*anti-hfq*OE) and the control plasmid pMMB207C (here named pMMB) were introduced via electroporation into wt *L. pneumophila* Paris strain. For overexpression, IPTG (0.5 mM) was added at an OD_600_ of 0.8.

### Sequencing of the *Δhfq* mutant strain.

For whole-genome sequencing, paired-end sequences and a read length of 100 bases were obtained from an Illumina HiSeq platform (Biomics pole Institut Pasteur). Sequence reads were mapped to a reference genome using SMALT v0.7.4, and single nucleotide polymorphisms (SNPs) were searched for using a standard approach.

### *A*. *castellanii* and THP-1 infection assay.

Infection of *A. castellanii* with *L. pneumophila* Paris and its derivatives was done as described previously (49). In brief, *A. castellanii* were washed once with infection buffer (PYG 712 medium without proteose peptone, glucose, and yeast extract) and seeded at a density of 4 × 10^6^ cells per 25-cm^2^ flask. Wild-type and mutant strains of *L. pneumophila* were grown on BCYE agar to stationary phase, diluted in infection buffer, and mixed with *A. castellanii* at a multiplicity of infection (MOI) of 0.1 or 1 (as indicated in the figure legends). Intracellular multiplication was monitored by plating a 100-μl sample that was centrifuged at 14,500 rpm and vortexed to break up amoeba, at different time points on BCYE plates. The number of bacteria recovered was counted as CFU. In THP-1 cell infection assays, cells were seeded in 12-well tissue culture trays (TTP) at a density of 2 × 10^5^ cells/well. THP-1 cells were pretreated with 10 to 8 M phorbol 12-myristate 13-acetate (PMA) (Sigma) for 72 h to induce differentiation into macrophage-like adherent cells. Stationary-phase *L. pneumophila* bacteria were resuspended in serum-free medium and added to cells at an MOI of 10. After 2 h of incubation, cells were washed with phosphate-buffered saline (PBS) before incubation with serum-free medium. At 2, 24, 48, and 72 h, the supernatant was collected and the cells were lysed with PBS–0.1% Triton X-100. The infection efficiency was monitored by determining the CFU of the different *L. pneumophila* strains recovered on BCYE agar plates. Each infection was carried out in triplicate.

### RNA isolation and Northern blot analysis.

Total RNA was extracted as previously described (50). Wild-type and mutant *L. pneumophila* Paris strains were grown in BYE medium and harvested for RNA isolation at exponential phase (OD_600_ of 1.0 and 2.0) and postexponential phase (OD_600_ of 3 and 4). Total RNA was treated with DNase I and purified using columns (Qiagen). Ten micrograms of total RNA isolated from different conditions (see above) were size separated on 10% denaturing polyacrylamide gels containing 8 M urea (Bio-Rad) and transferred onto positively charged nylon membranes (BrightStar-Plus; Ambion). The membranes were photographed under UV light to capture ethidium bromide staining of rRNA bands for loading controls. RNA was cross-linked to membranes by exposure to UV light for 2 min, and membranes were prehybridized in Ultrahyb buffer (catalog no. AM8670; Ambion) for 1 h. RNA probes radioactively labeled with [α-^33^P]UTP (catalog no. BLU007X500UC; PerkinElmer) were generated using the T7 Maxiscript kit (catalog no. AM1314; Ambion), and PCR templates were amplified from genomic DNA using primers listed in Table 2. The membrane was then hybridized at 65°C by adding the radiolabeled probes overnight. Blots were washed twice at the hybridization temperature in 2× SSC–0.1% SDS (1× SSC is 0.15 M NaCl plus 0.015 M sodium citrate) and then washed twice in 0.1× SSC–0.1% SDS. Membranes were wrapped in Saran Wrap and subsequently used to expose films (catalog no. 28906844; GE Healthcare).

### RNA isolation, labeling, and microarray hybridization.

For total RNA extraction, wild-type Paris and the *Δhfq* mutant strains were grown in BYE medium *in vitro* and harvested for RNA isolation at postexponential growth phase (OD_600_ of 4). For *in vivo* experiments, *A. castellanii* amoebae were infected with wt or *Δhfq* mutant at an MOI of 100 as described above. Cells were cultivated at 20°C and harvested for RNA isolation after 96 h. RNA was prepared in biological triplicates for *in vitro* and biological duplicates for *in vivo* experiments as described above, and all samples were hybridized twice to the microarrays (dye swap). RNA was reverse transcribed with Superscript indirect cDNA kit (Invitrogen) and labeled with Cy5 or Cy3 (Amersham Biosciences, Inc.) according to the supplier’s instructions. The design of microarrays containing gene-specific 70-mer oligonucleotides based on all predicted genes of the genome of *L. pneumophila* strain Paris (CR628336) and its plasmid (CR628338) was previously described (39). Hybridization was performed following the manufacturers’ recommendations (Corning) using 250 pmol of Cy3- and Cy5-labeled cDNA. Slides were scanned on a GenePix 4000A scanner (Axon Instruments). Laser power and/or the photomultiplier tube (PMT) were adjusted to balance the two channels, and the resulting files were analyzed using GenePix Pro 4.0 software. Spots were excluded from analysis in case of high local background fluorescence, slide abnormalities, or weak intensity.

Data normalization and differential analysis were conducted using the R software (http://www.R-project.org). No background subtraction was performed, but a careful graphical examination of all the slides was conducted to ensure a homogeneous, low-level background in both channels. A loess normalization (51) was performed on a slide-by-slide basis (BioConductor package marray; https://www.bioconductor.org/packages/release/bioc/html/marray.html). Differential analysis was carried out separately for each comparison between two time points, using the VM method (VarMixt package [52]), together with the Benjamini and Yekutieli *P* value adjustment method (53). Empty and flagged spots were excluded from the data set, and only genes with no missing values for the comparison of interest were analyzed.

### Determination of RNA half-life and quantitative RT-PCR.

Wild-type and RNase III gene deletion mutant strains of *L. pneumophila* were grown to an OD_600_ of 2.5 in BYE medium. Cells were subsequently treated with rifampin (final concentration of 500 μg/ml). Aliquots were removed at time zero (just before treatment) or after 5, 10, or 20 min of treatment. Cells were harvested by centrifugation in a tabletop centrifuge at 13,000 rpm for 1 min. Pellets were flash frozen in liquid nitrogen, and subsequently RNA was isolated as described above. Quantitative reverse transcription-PCR (qPCR) was then performed as described previously (39) at cDNA concentrations ranging from 5 ng to 5 × 10^−3^ ng. Primers used are listed in Table 2. Primer efficiencies were evaluated by generating a standard curve with serial dilutions, which indicated an efficiency of 90% to 110% for all primers used. The specificity of the amplified product and primer dimer formation was verified for each primer set by the presence of a single peak in a disassociation step carried out after each run. The absence of contaminating DNA was verified using control samples for each RNA sample for which no prior reverse transcription reaction had been carried out. Fold changes were calculated using the ΔΔ*C*_*T*_ method. Values represent mean values of three biological replicate experiments ± standard deviations (SD), normalized to the 16S loading controls.

### Western blot analysis.

Samples were denatured at 90°C for 10 min and separated on a 4 to 20% gradient SDS-PAGE gel (Bio-Rad) and transferred using a Trans-Blot Turbo transfer system (Bio-Rad). The membrane was stained with black amide or red ponceau solutions for loading controls and blocked in 1.2% bovine serum albumin (BSA) in Tris-buffered saline with Tween 20 (TBS-Tween) for 1 h at room temperature. Membranes were incubated overnight at 4°C with anti-Hfq or anti-FlaA primary antibodies that we generated. Briefly, Hfq and FlaA 6×His protein production was induced at an OD_600_ of 0.5 by 0.4 mM IPTG at 37°C for 4 h. Hfq and FlaA-6x-His proteins were purified using nickel-nitrilotriacetic acid (Ni-NTA) agarose beads and a Poly-Prep chromatography column. The resulting proteins were injected into rabbits, and crude sera were recovered 90 days later (Thermo Fisher Custom Antibody Services). Specific immunoglobulins were purified from serum samples by using a 1.0-ml HiTrap affinity NHS column (GE Healthcare) according to the manufacturer’s instructions. The antibody specificity and purity were assessed by Western blotting against the purified proteins. Membranes were incubated overnight at 4°C with Hfq or FlaA primary antibodies (diluted 1:2,000). The membranes were washed three times for 5 min each time in TBS–0.5% Tween at room temperature. The membrane was incubated for 1 h at room temperature with the secondary antibody, horseradish peroxidase (HRP)-labeled anti-rabbit (Dako) in TBS–0.5% Tween before the membrane was washed as described above. Signals were visualized using the ECL2 prime Western blot detection kit (Pierce) and the G-Box imaging system (Syngene).

### Rapid amplification of the 3′ end of cDNA (3′ RACE).

Amplification of the 3′-end region of anti*-hfq* was performed using total RNA purified from wt *L. pneumophila* Paris strain in the early exponential growth phase (OD_600_ of 2) as described above. Total RNA was treated with DNase I (Roche), incubated at 37°C with 10 U tobacco acid pyrophosphatase (TAP) (Epicentre) as previously described (28), subjected to phenol-chloroform-isoamyl alcohol (IAA) extraction (25:24:1), and precipitated overnight at −20°C with 10% 3 M sodium acetate (pH 5.2), 2% glycogen (20 mg/ml), and 2.5 volume of ethanol. For the 3′ adapter ligation, a mix of 3′ RNA adapters P-UCGUAUGCCGUCUUCUGCUUG-UidT (100 μM) was ligated to the processed RNA using the T4 RNA ligase (Epicentre) according to the manufacturer’s instructions. cDNA was then synthesized as described above, and amplification (primers *anti-hfq*_3’RACE _F and *anti-hfq*_3’RACE_R) products were fractionated in a 2% agarose gel. After staining with ethidium bromide, the sole band obtained of about 100 nucleotides (nt) was cut from the gel and purified using the NucleoSpin Extract kit (Macherey-Nagel). The purified size-selected cDNA fragment was cloned into the pGEM-T Easy (Promega) plasmid, and the cloned fragment was sequenced.

### RNA *in vitro* transcription and labeling.

Anti-hfq (103-nt), *hfq* mRNA (335-nt), *lpp0644* (137-nt), and *hfq*OUT (180-nt) genes used for electrophoretic mobility shift assay (EMSA) gel mobility assays and *hfq* mRNA (128 nt) used for Northern blot analyses were amplified from bacterial DNA with primers containing the T7 promoter at the 5′ end (Table 2). The resulting fragments were used as the templates to produce *in vitro* RNA (MEGAscript T7 kit; Ambion) and radioactively labeled with [α-^33^P]UTP (PerkinElmer). The reaction mixture was incubated at 37°C for 30 min, and RNA was digested with Turbo DNase digestion (1 U, 15 min at 37°C) and purified using the Illustra Micro-Spin G-25 columns (GE Healthcare) according to the supplier’s protocol.

### EMSA gel mobility assay.

RNA-RNA binding assays were performed to assess the binding affinity of the Anti-hfq transcript and *hfq* mRNA, *hfq*OUT (spanning the nucleotide sequence 78 to 255 and missing the 5′ UTR and the first 26 codons of the *hfq* transcript), and *lpp0644* as a control. Briefly, 25 nM anti*-hfq*, together with 0, 10, 15, 30, or 50 nM *hfq* full transcript or 0, 15, 30, or 50 nM *hfq*OUT probes was incubated with buffer containing 10 mM Tris-Cl (pH 8.3) and 0.1 mM EDTA, denatured at 70°C for 5 min, and cooled down for 15 min at room temperature. *In vitro* formation of complexes between Hfq and *hfq* mRNA or Anti-hfq sRNA (25 nM) *in vitro* was analyzed by EMSA using 0.05, 0.08, 0.1, 0.16, 0.22, 0.27, 0.5, and 1 μM His-tagged Hfq (Hfq6XHis) and supplemented with 5× structure buffer (50 mM Tris-HCl [pH 8.0], 250 mM NaCl, 250 mM KCl, 200 ng/ml tRNA) and incubated at 37°C for 15 min.

For the formation of ternary complexes, 25 nM radioactively labeled Anti-hfq and 25 nM cold *hfq* mRNA probes were incubated alone or with 1 μM His-tagged Hfq (Hfq6XHis) protein for 0.5, 1.5, or 2.5 min and supplemented with 5× structure buffer (50 mM Tris-HCl [pH 8.0], 250 mM NaCl, 250 mM KCl, 200 ng/ml tRNA). For a control, 25 nM radioactively labeled Anti-hfq or *hfq* mRNA probes were incubated with 0.5 and 1 μM (Hfq6XHis) for 10 min at room temperature. Prior to loading, reactions were mixed with native loading buffer, and samples were loaded onto 6 or 8% polyacrylamide 1× Tris-acetate-EDTA gel in 1× Tris-acetate EDTA running buffer. Following electrophoresis at 4°C, the gels were wrapped in Saran Wrap and subsequently exposed to films (GE Healthcare).

## References

[1] Waters LS, Storz G 2009 Regulatory RNAs in bacteria. Cell 136:615–628. doi:10.1016/j.cell.2009.01.043. 19239884 PMC3132550

[2] Caldelari I, Chao Y, Romby P, Vogel J 2013 RNA-mediated regulation in pathogenic bacteria. Cold Spring Harb Perspect Med 3:a010298. doi:10.1101/cshperspect.a010298. 24003243 PMC3753719

[3] Gottesman S, Storz G 2011 Bacterial small RNA regulators: versatile roles and rapidly evolving variations. Cold Spring Harb Perspect Biol 3:a003798. doi:10.1101/cshperspect.a003798. 20980440 PMC3225950

[4] Møller T, Franch T, Højrup P, Keene DR, Bächinger HP, Brennan RG, Valentin-Hansen P 2002 Hfq: a bacterial Sm-like protein that mediates RNA-RNA interaction. Mol Cell 9:23–30. doi:10.1016/S1097-2765(01)00436-1. 11804583

[5] Franze de Fernandez MT, Eoyang L, August JT 1968 Factor fraction required for the synthesis of bacteriophage Qbeta-RNA. Nature 219:588–590. doi:10.1038/219588a0. 4874917

[6] Updegrove TB, Zhang A, Storz G 2016 Hfq: the flexible RNA matchmaker. Curr Opin Microbiol 30:133–138. doi:10.1016/j.mib.2016.02.003. 26907610 PMC4821791

[7] Chao Y, Vogel J 2010 The role of Hfq in bacterial pathogens. Curr Opin Microbiol 13:24–33. doi:10.1016/j.mib.2010.01.001. 20080057

[8] Tsui HC, Leung HC, Winkler ME 1994 Characterization of broadly pleiotropic phenotypes caused by an hfq insertion mutation in Escherichia coli K-12. Mol Microbiol 13:35–49. doi:10.1111/j.1365-2958.1994.tb00400.x. 7984093

[9] Vogel J, Luisi BF 2011 Hfq and its constellation of RNA. Nat Rev Microbiol 9:578–589. doi:10.1038/nrmicro2615. 21760622 PMC4615618

[10] Sittka A, Lucchini S, Papenfort K, Sharma CM, Rolle K, Binnewies TT, Hinton JC, Vogel J 2008 Deep sequencing analysis of small noncoding RNA and mRNA targets of the global post-transcriptional regulator, Hfq. PLoS Genet 4:e1000163. doi:10.1371/journal.pgen.1000163. 18725932 PMC2515195

[11] Wurtzel O, Yoder-Himes DR, Han K, Dandekar AA, Edelheit S, Greenberg EP, Sorek R, Lory S 2012 The single-nucleotide resolution transcriptome of *Pseudomonas aeruginosa* grown in body temperature. PLoS Pathog 8:e1002945. doi:10.1371/journal.ppat.1002945. 23028334 PMC3460626

[12] Nuss AM, Heroven AK, Waldmann B, Reinkensmeier J, Jarek M, Beckstette M, Dersch P 2015 Transcriptomic profiling of *Yersinia pseudotuberculosis* reveals reprogramming of the Crp regulon by temperature and uncovers Crp as a master regulator of small RNAs. PLoS Genet 11:e1005087. doi:10.1371/journal.pgen.1005087. 25816203 PMC4376681

[13] Sahr T, Rusniok C, Dervins-Ravault D, Sismeiro O, Coppee JY, Buchrieser C 2012 Deep sequencing defines the transcriptional map of *L. pneumophila* and identifies growth phase-dependent regulated ncRNAs implicated in virulence. RNA Biol 9:503–519. doi:10.4161/rna.20270. 22546937

[14] Jørgensen MG, Nielsen JS, Boysen A, Franch T, Møller-Jensen J, Valentin-Hansen P 2012 Small regulatory RNAs control the multi-cellular adhesive lifestyle of *Escherichia coli*. Mol Microbiol 84:36–50. doi:10.1111/j.1365-2958.2012.07976.x. 22250746

[15] Beisel CL, Storz G 2011 Discriminating tastes: physiological contributions of the Hfq-binding small RNA Spot 42 to catabolite repression. RNA Biol 8:766–770. doi:10.4161/rna.8.5.16024. 21788732 PMC3256354

[16] Lenz DH, Mok KC, Lilley BN, Kulkarni RV, Wingreen NS, Bassler BL 2004 The small RNA chaperone Hfq and multiple small RNAs control quorum sensing in *Vibrio harveyi* and *Vibrio cholerae*. Cell 118:69–82. doi:10.1016/j.cell.2004.06.009. 15242645

[17] Coornaert A, Lu A, Mandin P, Springer M, Gottesman S, Guillier M 2010 MicA sRNA links the PhoP regulon to cell envelope stress. Mol Microbiol 76:467–479. doi:10.1111/j.1365-2958.2010.07115.x. 20345657 PMC2925231

[18] Sobrero P, Valverde C 2012 The bacterial protein Hfq: much more than a mere RNA-binding factor. Crit Rev Microbiol 38:276–299. doi:10.3109/1040841X.2012.664540. 22435753

[19] Cazalet C, Rusniok C, Brüggemann H, Zidane N, Magnier A, Ma L, Tichit M, Jarraud S, Bouchier C, Vandenesch F, Kunst F, Etienne J, Glaser P, Buchrieser C 2004 Evidence in the *Legionella pneumophila* genome for exploitation of host cell functions and high genome plasticity. Nat Genet 36:1165–1173. doi:10.1038/ng1447. 15467720

[20] McNealy TL, Forsbach-Birk V, Shi C, Marre R 2005 The Hfq homolog in *Legionella pneumophila* demonstrates regulation by LetA and RpoS and interacts with the global regulator CsrA. J Bacteriol 187:1527–1532. doi:10.1128/JB.187.4.1527-1532.2005. 15687220 PMC545622

[21] Molofsky AB, Swanson MS 2004 Differentiate to thrive: lessons from the *Legionella pneumophila* life cycle. Mol Microbiol 53:29–40. doi:10.1111/j.1365-2958.2004.04129.x. 15225301 PMC13218203

[22] Edwards RL, Jules M, Sahr T, Buchrieser C, Swanson MS 2010 The *Legionella pneumophila* LetA/LetS two-component system exhibits rheostate-like behavior. Infect Immun 78:2571–2583. doi:10.1128/IAI.01107-09. 20351136 PMC2876543

[23] Hammer BK, Tateda ES, Swanson MS 2002 A two-component regulator induces the transmission phenotype of stationary-phase *Legionella pneumophila*. Mol Microbiol 44:107–118. doi:10.1046/j.1365-2958.2002.02884.x. 11967072 PMC13220096

[24] Lynch D, Fieser N, Glöggler K, Forsbach-Birk V, Marre R 2003 The response regulator LetA regulates the stationary-phase stress response in *Legionella pneumophila* and is required for efficient infection of *Acanthamoeba* *castellanii*. FEMS Microbiol Lett 219:241–248. doi:10.1016/S0378-1097(03)00050-8. 12620627

[25] Fettes PS, Forsbach-Birk V, Lynch D, Marre R 2001 Overexpression of a *Legionella pneumophila* homologue of the *E. coli* regulator *csrA* affects cell size, flagellation, and pigmentation. Int J Med Microbiol 291:353–360. doi:10.1078/1438-4221-00141. 11727819

[26] Molofsky AB, Swanson MS 2003 *Legionella pneumophila* CsrA is a pivotal repressor of transmission traits and activator of replication. Mol Microbiol 50:445–461. doi:10.1046/j.1365-2958.2003.03706.x. 14617170 PMC13227487

[27] Rasis M, Segal G 2009 The LetA-RsmYZ-CsrA regulatory cascade, together with RpoS and PmrA, post-transcriptionally regulates stationary phase activation of *Legionella pneumophila* Icm/Dot effectors. Mol Microbiol 72:995–1010. doi:10.1111/j.1365-2958.2009.06705.x. 19400807

[28] Sahr T, Brüggemann H, Jules M, Lomma M, Albert-Weissenberger C, Cazalet C, Buchrieser C 2009 Two small ncRNAs jointly govern virulence and transmission in *Legionella pneumophila*. Mol Microbiol 72:741–762. doi:10.1111/j.1365-2958.2009.06677.x. 19400772 PMC2888818

[29] D’Auria G, Jiménez-Hernández N, Peris-Bondia F, Moya A, Latorre A 2010 *Legionella pneumophila* pangenome reveals strain-specific virulence factors. BMC Genomics 11:181. doi:10.1186/1471-2164-11-181. 20236513 PMC2859405

[30] Gomez-Valero L, Rusniok C, Jarraud S, Vacherie B, Rouy Z, Barbe V, Medigue C, Etienne J, Buchrieser C 2011 Extensive recombination events and horizontal gene transfer shaped the *Legionella pneumophila* genomes. BMC Genomics 12:536. doi:10.1186/1471-2164-12-536. 22044686 PMC3218107

[31] Schroeder GN, Petty NK, Mousnier A, Harding CR, Vogrin AJ, Wee B, Fry NK, Harrison TG, Newton HJ, Thomson NR, Beatson SA, Dougan G, Hartland EL, Frankel G 2010 *Legionella pneumophila* strain 130b possesses a unique combination of type IV secretion systems and novel Dot/Icm secretion system effector proteins. J Bacteriol 192:6001–6016. doi:10.1128/JB.00778-10. 20833813 PMC2976443

[32] David S, Rusniok C, Mentasti M, Gomez-Valero L, Harris SR, Lechat P, Lees J, Ginevra C, Glaser P, Ma L, Bouchier C, Underwood A, Jarraud S, Harrison TG, Parkhill J, Buchrieser C 2016 Multiple major disease-associated clones of *Legionella pneumophila* have emerged recently and independently. Genome Res 26:1555–1564. doi:10.1101/gr.209536.116. 27662900 PMC5088597

[33] Gomez-Valero L, Rusniok C, Rolando M, Neou M, Dervins-Ravault D, Demirtas J, Rouy Z, Moore RJ, Chen H, Petty NK, Jarraud S, Etienne J, Steinert M, Heuner K, Gribaldo S, Médigue C, Glöckner G, Hartland EL, Buchrieser C 2014 Comparative analyses of Legionella species identifies genetic features of strains causing Legionnaires’ disease. Genome Biol 15:505. doi:10.1186/PREACCEPT-1086350395137407. 25370836 PMC4256840

[34] Trigui H, Dudyk P, Sum J, Shuman HA, Faucher SP 2013 Analysis of the transcriptome of *Legionella pneumophila* *hfq* mutant reveals a new mobile genetic element. Microbiology 159:1649–1660. doi:10.1099/mic.0.067983-0. 23728622 PMC5972305

[35] Cirillo SL, Lum J, Cirillo JD 2000 Identification of novel loci involved in entry by *Legionella pneumophila*. Microbiology 146:1345–1359. doi:10.1099/00221287-146-6-1345. 10846213

[36] Zuker M 2003 Mfold web server for nucleic acid folding and hybridization prediction. Nucleic Acids Res 31:3406–3415. doi:10.1093/nar/gkg595. 12824337 PMC169194

[37] Christiansen JK, Nielsen JS, Ebersbach T, Valentin-Hansen P, Søgaard-Andersen L, Kallipolitis BH 2006 Identification of small Hfq-binding RNAs in *Listeria monocytogenes*. RNA 12:1383–1396. doi:10.1261/rna.49706. 16682563 PMC1484441

[38] Sonnleitner E, Schuster M, Sorger-Domenigg T, Greenberg EP, Bläsi U 2006 Hfq-dependent alterations of the transcriptome profile and effects on quorum sensing in *Pseudomonas aeruginosa*. Mol Microbiol 59:1542–1558. doi:10.1111/j.1365-2958.2006.05032.x. 16468994

[39] Brüggemann H, Hagman A, Jules M, Sismeiro O, Dillies MA, Gouyette C, Kunst F, Steinert M, Heuner K, Coppée JY, Buchrieser C 2006 Virulence strategies for infecting phagocytes deduced from the in vivo transcriptional program of *Legionella pneumophila*. Cell Microbiol 8:1228–1240. doi:10.1111/j.1462-5822.2006.00703.x. 16882028

[40] Duprey A, Reverchon S, Nasser W 2014 Bacterial virulence and Fis: adapting regulatory networks to the host environment. Trends Microbiol 22:92–99. doi:10.1016/j.tim.2013.11.008. 24370464

[41] Ishikawa H, Otaka H, Maki K, Morita T, Aiba H 2012 The functional Hfq-binding module of bacterial sRNAs consists of a double or single hairpin preceded by a U-rich sequence and followed by a 3′ poly(U) tail. RNA 18:1062–1074. doi:10.1261/rna.031575.111. 22454537 PMC3334693

[42] Sauer E, Schmidt S, Weichenrieder O 2012 Small RNA binding to the lateral surface of Hfq hexamers and structural rearrangements upon mRNA target recognition. Proc Natl Acad Sci U S A 109:9396–9401. doi:10.1073/pnas.1202521109. 22645344 PMC3386104

[43] Vecerek B, Moll I, Bläsi U 2005 Translational autocontrol of the *Escherichia coli* *hfq* RNA chaperone gene. RNA 11:976–984. doi:10.1261/rna.2360205. 15872186 PMC1370782

[44] Zusman T, Speiser Y, Segal G 2014 Two Fis regulators directly repress the expression of numerous effector-encoding genes in *Legionella pneumophila*. J Bacteriol 196:4172–4183. doi:10.1128/JB.02017-14. 25225276 PMC4248873

[45] Feeley JC, Gibson RJ, Gorman GW, Langford NC, Rasheed JK, Mackel DC, Baine WB 1979 Charcoal-yeast extract agar: primary isolation medium for *Legionella pneumophila*. J Clin Microbiol 10:437–441. 393713 10.1128/jcm.10.4.437-441.1979 PMC273193

[46] Hindré T, Brüggemann H, Buchrieser C, Héchard Y 2008 Transcriptional profiling of *Legionella pneumophila* biofilm cells and the influence of iron on biofilm formation. Microbiology 154:30–41. doi:10.1099/mic.0.2007/008698-0. 18174123

[47] Buchrieser C, Charpentier X 2013 Induction of competence for natural transformation in *Legionella pneumophila* and exploitation for mutant construction. Methods Mol Biol 954:183–195. doi:10.1007/978-1-62703-161-5_9. 23150395

[48] Weber SS, Ragaz C, Hilbi H 2009 The inositol polyphosphate 5-phosphatase OCRL1 restricts intracellular growth of *Legionella*, localizes to the replicative vacuole and binds to the bacterial effector LpnE. Cell Microbiol 11:442–460. doi:10.1111/j.1462-5822.2008.01266.x. 19021631

[49] Rolando M, Escoll P, Nora T, Botti J, Boitez V, Bedia C, Daniels C, Abraham G, Stogios PJ, Skarina T, Christophe C, Dervins-Ravault D, Cazalet C, Hilbi H, Rupasinghe TW, Tull D, McConville MJ, Ong SY, Hartland EL, Codogno P, Levade T, Naderer T, Savchenko A, Buchrieser C 2016 Legionella pneumophila S1P-lyase targets host sphingolipid metabolism and restrains autophagy. Proc Natl Acad Sci U S A 113:1901–1906. doi:10.1073/pnas.1522067113. 26831115 PMC4763766

[50] Milohanic E, Glaser P, Coppée JY, Frangeul L, Vega Y, Vázquez-Boland JA, Kunst F, Cossart P, Buchrieser C 2003 Transcriptome analysis of *Listeria monocytogenes* identifies three groups of genes differently regulated by PrfA. Mol Microbiol 47:1613–1625. doi:10.1046/j.1365-2958.2003.03413.x. 12622816

[51] Yang YH, Dudoit S, Luu P, Lin DM, Peng V, Ngai J, Speed TP 2002 Normalization for cDNA microarray data: a robust composite method addressing single and multiple slide systematic variation. Nucleic Acids Res 30:e15. doi:10.1093/nar/30.4.e15. 11842121 PMC100354

[52] Delmar P, Robin S, Daudin JJ 2005 VarMixt: efficient variance modelling for the differential analysis of replicated gene expression data. Bioinformatics 21:502–508. doi:10.1093/bioinformatics/bti023. 15374871

[53] Reiner A, Yekutieli D, Benjamini Y 2003 Identifying differentially expressed genes using false discovery rate controlling procedures. Bioinformatics 19:368–375. doi:10.1093/bioinformatics/btf877. 12584122

[54] Rolando M, Sanulli S, Rusniok C, Gomez-Valero L, Bertholet C, Sahr T, Margueron R, Buchrieser C 2013 Legionella pneumophila effector RomA uniquely modifies host chromatin to repress gene expression and promote intracellular bacterial replication. Cell Host Microbe 13:395–405. doi:10.1016/j.chom.2013.03.004. 23601102

[55] Weber SS, Ragaz C, Reus K, Nyfeler Y, Hilbi H 2006 *Legionella pneumophila* exploits PI(4)P to anchor secreted effector proteins to the replicative vacuole. PLoS Pathog 2:e46. doi:10.1371/journal.ppat.0020046. 16710455 PMC1463015

