# Supplementary figures and images for: A Unique cis-Encoded Small Noncoding RNA Is Regulating Legionella pneumophila Hfq Expression in a Life Cycle-Dependent Manner
Source: mBio. 2017 Jan 10;8(1):e02182-16. doi: 10.1128/mBio.02182-16 (PMC5225317; doi:10.1128/mBio.02182-16)

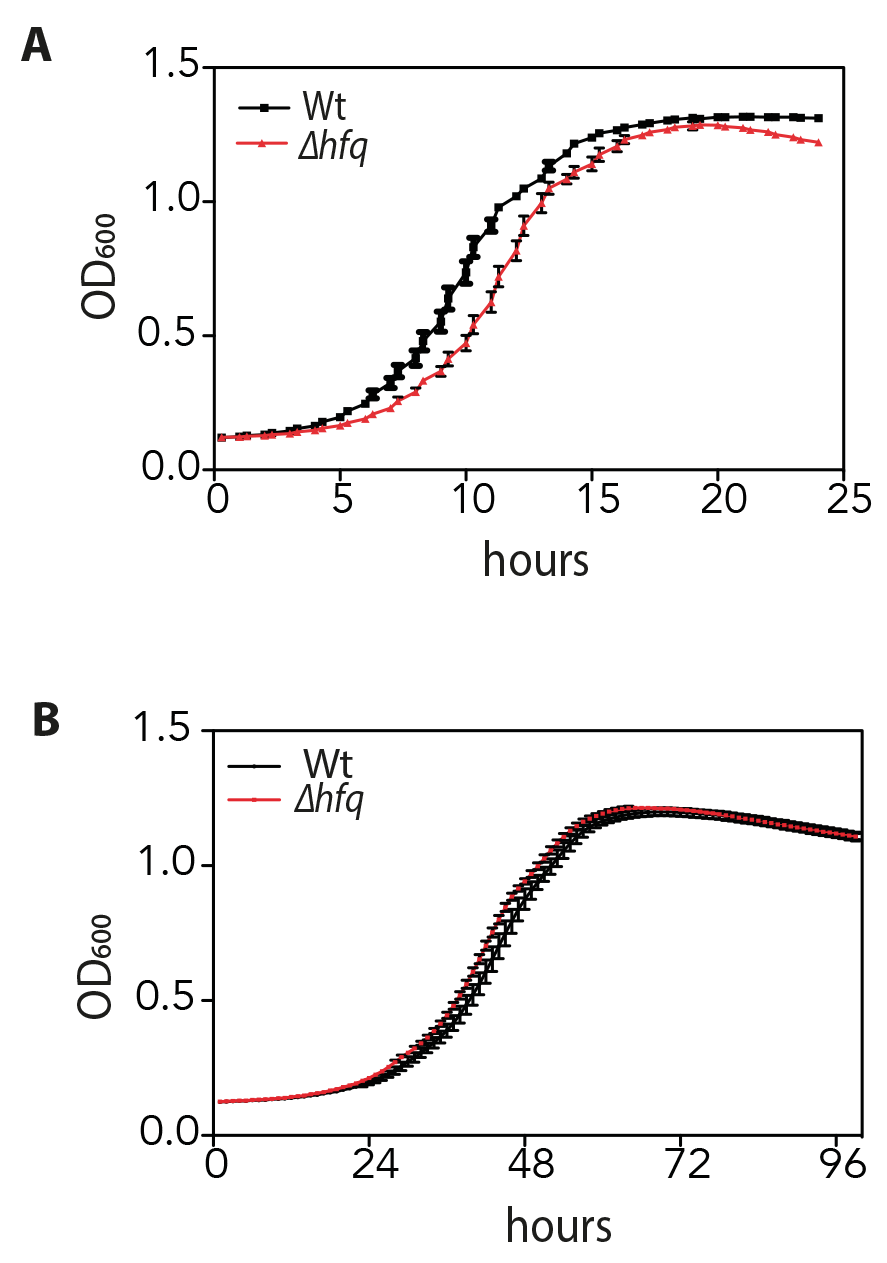

Supplement: FIG S1 [file mbo006163135sf1.tif]

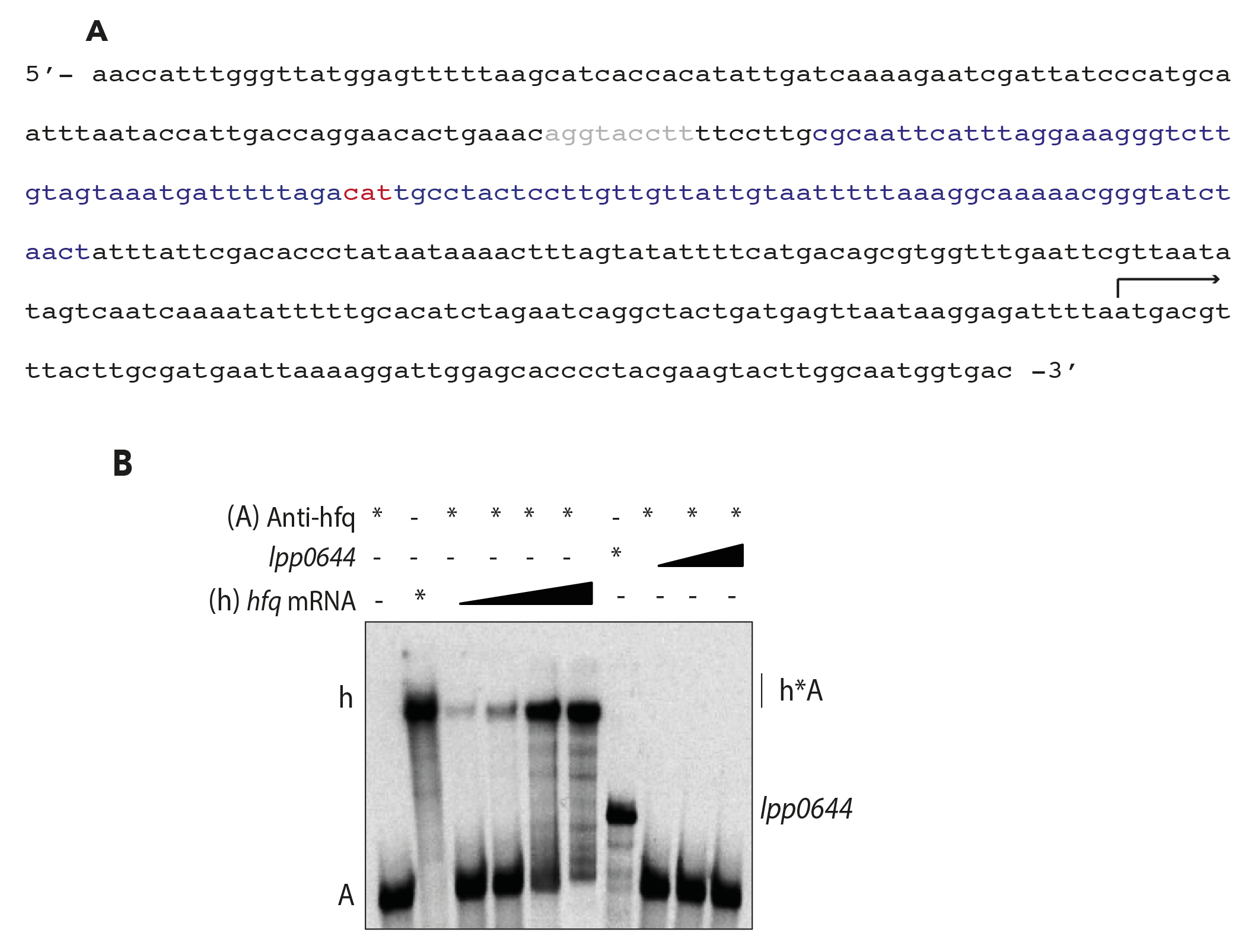

Supplement: FIG S2 [file mbo006163135sf2.tif]
