# Supplementary material for: A Unique cis-Encoded Small Noncoding RNA Is Regulating Legionella pneumophila Hfq Expression in a Life Cycle-Dependent Manner
Source: mBio. 2017 Jan 10;8(1):e02182-16. doi: 10.1128/mBio.02182-16 (PMC5225317; doi:10.1128/mBio.02182-16)
Supplement: TABLE S1 [file mbo006163135st1.docx]

**Table S1: Differentially expressed genes in *in vitro* analyses of wt and *Δhfq* grown in BYE broth at 37°C** **until OD_600_ 4 (PE phase)**

Up-regulated in the ***Δ****hfq* strain (p<0.05, 8 up-regulated also *in vivo* analyses)

| **gene.ID** | **description** | **FC** |
| --- | --- | --- |
| *lpp0460* | hypothetical protein | 2,04 |
| *lpp0973* | pantothenate kinase type III (putative Bvg accessory factor family protein) | 2,32 |
| *lpp1229* | flagellar biosynthesis protein FlgG | 3,29 |
| *lpp1230* | flagellar L-ring protein precursor FlgH | 3,36 |
| *lpp1231* | flagellar P-ring protein precursor FlgI | 2,01 |
| *lpp1291* | flagellar protein FliS | 2,49 |
| *lpp1292* | flagellar hook-associated protein 2 (flagellar capping protein) | 2,49 |
| *lpp1974* | uncharacterized bacterial polysaccharide deacetylases, catalytic NodB homology domain | 2,26 |
| *lpp2350* | chemiosmotic efflux system C protein A | 3,09 |
| *lpp2351* | chemiosmotic efflux system protein A-like protein | 2,21 |
| *lpp2353* | chemiosmotic efflux system C protein C | 5,49 |
| *lpp2354* | domain of unknown function (DUF4156) | 4,63 |
| *lpp2693* | enhanced entry protein EnhB - Sel1-like repeats protein | 2,99 |
| *lpp2694* | enhanced entry protein EnhA - L,D-transpeptidase catalytic domain, | 2,42 |
| *lpp2988* | lytic murein transglycosylase, SLT domain | 3,43 |

Down-regulated in the ***∆****hfq* strain (p<0.05, 1 down-regulated also *in vivo* analyses)

| **gene.ID** | **description** | **FC** |
| --- | --- | --- |
| *lpp0009* | host factor-1 protein Hfq | 0,07 |
| *lpp0679* | StaR-like protein, TPR-domain - eukaryotic-like protein | 0,48 |
| *sRNA* | RsmX | 0,48 |
