# Supplementary material for: A Unique cis-Encoded Small Noncoding RNA Is Regulating Legionella pneumophila Hfq Expression in a Life Cycle-Dependent Manner
Source: mBio. 2017 Jan 10;8(1):e02182-16. doi: 10.1128/mBio.02182-16 (PMC5225317; doi:10.1128/mBio.02182-16)
Supplement: TABLE S2 [file mbo006163135st2.docx]

**Table S2: Differentially expressed genes according to transcriptome *in vivo* analyses of wt and *Δhfq* grown for 96h at 20°C within *A. castelanii***

Up-regulated in the ***Δ****hfq* strain (p<0.05)

| **gene.ID** | | **description** | **FC** |
| --- | --- | --- | --- |
| *lpp0008* | substrate of the Dot/Icm secretion system RavA | | 1,91 |
| *lpp0122* | protein of unknown function | | 1,88 |
| *lpp0257* | chitin-binding protein CbpD | | 1,87 |
| *lpp0381* | preprotein translocase secE subunit | | 1,93 |
| *lpp0384* | 50S ribosomal protein L1 | | 2,07 |
| *lpp0385* | 50S ribosomal subunit protein L1 | | 2,19 |
| *lpp0386* | 50S ribosomal subunit protein L7/L12 | | 2,40 |
| *lpp0389* | 30S ribosomal protein S12 | | 2,03 |
| *lpp0392* | translation elongation factor Tu | | 2,39 |
| *lpp0393* | 30S ribosomal subunit protein S1 | | 2,05 |
| *lpp0394* | 50S ribosomal subunit protein L3 | | 1,88 |
| *lpp0396* | 50S ribosomal subunit protein L23 | | 2,02 |
| *lpp0398* | 30S ribosomal subunit protein S19 | | 2,08 |
| *lpp0399* | 50S ribosomal subunit protein L22 | | 1,88 |
| *lpp0400* | 30S ribosomal protein S3 | | 2,19 |
| *lpp0401* | 50S ribosomal protein L16 | | 2,26 |
| *lpp0402* | 50S ribosomal subunit protein L29 | | 1,96 |
| *lpp0403* | 30S ribosomal protein S17 | | 1,98 |
| *lpp0404* | 50S ribosomal protein L14 | | 2,14 |
| *lpp0406* | 50S ribosomal protein L5 | | 1,96 |
| *lpp0407* | 30S ribosomal protein S14 | | 2,46 |
| *lpp0408* | 30S ribosomal protein S8 | | 2,12 |
| *lpp0410* | 50S ribosomal subunit protein L18 | | 2,20 |
| *lpp0411* | 30S ribosomal subunit protein S5 | | 2,17 |
| *lpp0412* | 50S ribosomal subunit protein L3 | | 2,08 |
| *lpp0418* | 30S ribosomal subunit protein S4 | | 2,03 |
| *lpp0419* | DNA-directed RNA polymerase alpha chain | | 1,98 |
| *lpp0460* | hypothetical protein | | 2,65 |
| *lpp0466* | 30S ribosomal protein S16 | | 1,87 |
| *lpp0493* | cold shock-like protein CspD | | 2,47 |
| *lpp0544* | 50S ribosomal protein L28 | | 1,95 |
| *lpp0570* | outer membrane protein (OmpH-like) | | 2,10 |
| *lpp0605* | bacterial protein of unknown function (DUF945) | | 1,99 |
| *lpp0606* | global DNA-binding transcriptional regulator Fis1 | | 2,35 |
| *lpp0688* | substrate of the Dot/Icm secretion system | | 2,21 |
| *lpp0725* | predicted integral membrane protein (DUF2282) | | 2,08 |
| *lpp0742* | chaperonin 10 subunit, Cpn10 or GroES | | 1,96 |
| *lpp0743* | chaperonin 60, Cpn60 or GroEL | | 1,94 |
| *lpp0855* | macrophage infectivity potentiator Mip | | 2,52 |
| *lpp0972* | enhanced entry protein EnhA (L,D-transpeptidase catalytic domain) | | 2,56 |
| *lpp1146* | substrate of the Dot/Icm secretion system | | 2,46 |
| *lpp1207* | cold-shock protein (CSP) | | 2,24 |
| *lpp1229* | flagellar biosynthesis protein FlgG | | 1,86 |
| *lpp1230* | flagellar L-ring protein precursor FlgH | | 1,82 |
| *lpp1324* | global DNA-binding transcriptional regulator Fis2 | | 2,48 |
| *lpp1772* | hypothetical protein | | 2,07 |
| *lpp1805* | Com1-like membrane-associated immunoreactive protein, DsbA family | | 1,88 |
| *lpp1826* | DNA-binding protein HU-beta | | 2,82 |
| *lpp1958* | Legionella major outer membrane protein | | 2,45 |
| *lpp1974* | uncharacterized polysaccharide deacetylases, catalytic NodB homology domain | | 1,86 |
| *lpp2026* | peptidoglycan-associated lipoprotein, OmpA-like domain | | 2,07 |
| *lpp2164* | heme-binding protein Hbp | | 1,92 |
| *lpp2209* | hypothetical membrane protein | | 2,48 |
| *lpp2276* | substrate of the Dot/Icm secretion system | | 2,09 |
| *lpp2354* | domain of unknown function (DUF4156) | | 1,91 |
| *lpp2675* | papain-like C1 peptidase | | 1,93 |
| *lpp2689* | 30S ribosomal subunit protein S2 | | 2,08 |
| *lpp2692* | enhanced entry protein EnhC - Sel1-like repeats protein | | 1,83 |
| *lpp2693* | enhanced entry protein EnhB - Sel1-like repeats protein | | 1,97 |
| *lpp2694* | enhanced entry protein EnhA - L,D-transpeptidase catalytic domain, | | 1,68 |
| *lpp2768* | 50S ribosomal protein L35 | | 2,05 |
| *lpp2817* | 30S ribosomal protein S15 | | 1,94 |
| *lpp2866* | leucine aminopeptidase, Zn-peptidase M28 family | | 1,89 |
| *lpp2968* | hypothetical protein | | 1,95 |
| *lpp2988* | lytic murein transglycosylase, SLT domain | | 1,72 |
| *lpp3021* | hypothetical protein | | 2,09 |
| *lpp3031* | major outer membrane protein precursor | | 2,47 |
| *lpp3032* | major outer membrane protein precursor | | 3,24 |
| *lpp3033* | major outer membrane protein precursor | | 3,07 |

Down-regulated in the ***Δ****hfq* strain (p<0.05)

| **gene.ID** | | | **description** | | **FC** |  |
| --- | --- | --- | --- | --- | --- | --- |
| *lpp0009* | | host factor-1 protein Hfq | | | 0,19 |  |
| *lpp0034* | hypothetical protein | | | 0,52 | | |
| *lpp0045* | fatty acid hydroxylase | | | 0,52 | | |
| *lpp0845* | global regulator CsrA | | | 0,43 | | |
| *lpp1823* | hypothetical protein | | | 0,48 | | |
